# Antibiotic-Induced Genotoxicity: Molecular Mechanisms, Cytogenetic Damage, and Implications for Human Health

**DOI:** 10.3390/ijms27167460

**Published:** 2026-08-20

**Authors:** Ahmet Ali Berber, Esra Yıldız, Şefika Nur Demir, Nihan Akıncı Kenanoğlu, Nurcan Berber

**Affiliations:** 1 Vocational School of Health Services, Çanakkale Onsekiz Mart University, 17100 Canakkale, Türkiye; aberber@comu.edu.tr (A.A.B.); nberber@comu.edu.tr (N.B.); 2 Department of Biology, Faculty of Science, Sakarya University, 54050 Sakarya, Türkiye; 3 Department of Molecular Biology and Genetics, Faculty of Science, Ataturk University, 25240 Erzurum, Türkiye; sefikademir@atauni.edu.tr; 4 Department of Biology, Faculty of Science, Çanakkale Onsekiz Mart University, 17100 Canakkale, Türkiye; nakinci@comu.edu.tr

**Keywords:** antibiotic-induced genotoxicity, cytogenetic damage, mitochondrial dysfunction, DNA damage response, micronucleus assay, fluoroquinolones, pediatric antibiotic exposure, antimicrobial stewardship

## Abstract

Global antibiotic consumption continues to rise across pediatric and adult populations, while the genotoxic consequences of host eukaryotic exposure remain less systematically characterized than the parallel problem of antimicrobial resistance. Several lines of evidence, from molecular cytogenetics, redox biology, and systems toxicology, now permit a more mechanistically resolved synthesis of antibiotic-induced genome stress than was previously possible, although a substantial fraction of this evidence is preclinical and warrants cautious clinical extrapolation. This narrative review evaluates the molecular mechanisms, cytogenetic biomarkers, and translational implications of antibiotic-induced genotoxicity, with a primary focus on six clinically prominent classes (fluoroquinolones, nitroimidazoles, aminoglycosides, macrolides, β-lactams, and tetracyclines) and a brief extension to glycopeptides and glycylcyclines. We organize the evidence around three convergent mechanistic axes rather than around individual drugs. Accumulating evidence supports three intersecting off-target axes: (i) eukaryotic topoisomerase II interference, principally documented for fluoroquinolones; (ii) mitochondrial dysfunction, reflecting the evolutionary kinship between the mitoribosome and bacterial ribosomes; and (iii) inflammation-coupled redox stress, often amplified by microbiome perturbation. These pathways converge on a common spectrum of DNA lesions—including double-strand breaks, oxidatively modified bases, replication-fork stalling, and chromosomal mis-segregation) detected by complementary assays (CBMN-Cyt, comet, γH2AX, and oxidative and mitochondrial biomarkers). Pediatric, pregnant, geriatric, and oncology populations may represent biologically distinct susceptibility strata, although direct human evidence for several of these inferences remains limited. Causal inference is constrained by infection as a confounder, frequent use of supratherapeutic in vitro concentrations, reliance on immortalized cell lines that may not recapitulate primary-cell repair capacity, inter-laboratory variability across cytogenetic assays, and a marked scarcity of pediatric and pregnancy biomonitoring data. Most existing positive signals derive from preclinical models; clinically validated long-term outcomes, particularly carcinogenic endpoints, remain inconsistently demonstrated for most antibiotic classes outside metronidazole. Antibiotic-induced genotoxicity appears to be a measurable and mechanistically tractable dimension of drug safety, though its clinical magnitude in real-world exposure scenarios requires further investigation. Integrating multi-omics, microphysiological systems, single-cell genotoxicology, and AI-assisted prediction may improve risk resolution, particularly in vulnerable populations. We argue that antimicrobial stewardship discussions should consider host genome integrity alongside resistance, while remaining mindful that the mechanistic case currently outpaces clinical-endpoint validation.

## 1. Introduction

### 1.1. Indispensable Therapy, Imperfectly Characterized Safety

Antibiotics remain among the most consequential therapeutic interventions of the past century. Their continued indispensability across pediatric care, adult medicine, surgical prophylaxis, and management of immunocompromised hosts is rivalled by few other drug classes [1]. Between 2000 and 2015, global antibiotic consumption expressed in defined daily doses (DDDs) increased by 65%, from 21.1 to 34.8 billion DDDs, with the consumption rate rising 39% from 11.3 to 15.7 DDDs per 1000 inhabitants per day across 76 countries [2]. The increase was driven predominantly by low- and middle-income countries (LMICs), where consumption rates rose 77% (from 7.6 to 13.5 DDDs per 1000 inhabitants per day), while high-income countries (HICs) saw a modest 4% decrease (from 26.8 to 25.7 DDDs per 1000 inhabitants per day). Updated data for 2016–2023 indicate that this upward trajectory has continued: total consumption in 67 reporting countries rose a further 16.3% (from 29.5 to 34.3 billion DDDs), with estimated global consumption reaching 49.3 billion DDDs in 2023, and projected to increase by 52.3% to 75.1 billion DDDs by 2030 if current trends persist [3]. The COVID-19 pandemic temporarily reduced consumption across all income groups, but post-pandemic rebounds, particularly in middle-income countries, have restored the pre-pandemic growth trajectory. Available early-life antibiotic exposure is common: in the TEDDY cohort, 74–92% of children across six clinical centers in the United States and Europe received at least one antibiotic prescription during the first five years of life [4], and US data indicate that the average child receives approximately three antibiotic courses by age two [5].

The dominant scientific narrative around this trajectory has focused, understandably, on antimicrobial resistance. A parallel question has received less integrative attention: to what extent do antibiotics themselves perturb the host genome? The tension is genuine but not paradoxical. Antibiotics are indispensable for survival in the face of bacterial infection. At therapeutically relevant concentrations, they can also, in some experimental systems, affect eukaryotic DNA, mitochondria, and chromatin [6,7]. This is not an argument against antibiotic use where clinically indicated; it is a quantitative safety dimension whose mechanistic and translational characterization has lagged the resistance discussion.

### 1.2. Historical Recognition and Mechanistic Maturation

Concern about antibiotic-induced damage to eukaryotic nucleic acids is not new. Cytogenetic surveys from the 1970s reported elevated frequencies of chromosomal aberrations and sister chromatid exchanges in patients receiving nitroimidazoles, supporting the hypothesis that bioreductive activation is not strictly confined to anaerobic pathogens [8,9]. Around the same period, aminoglycoside ototoxicity was being linked, initially speculatively, to mitochondrial perturbation. That inference was later strengthened by the identification of the m.1555A>G mitochondrial 12S rRNA variant as a Mendelian susceptibility factor [10]. By the early 1990s, biochemical and cytogenetic studies had shown that fluoroquinolones, originally engineered to trap bacterial gyrase, can also inhibit eukaryotic topoisomerase II at concentrations achievable in some mammalian tissues, producing cleavable complexes that resemble those formed by established Topo II–poison chemotherapeutics, such as etoposide [11,12,13].

What has changed over the past decade is the resolution available for interrogating these phenomena. Standardization of the γH2AX immunoassay [14,15], maturation of the cytokinesis-block micronucleus cytome (CBMN-Cyt) assay under OECD Test Guideline 487 [16,17], expansion of the comet assay framework under OECD TG 489 [18], and the integration of mitochondrial bioenergetic profiling, single-cell transcriptomics, and high-content imaging have together moved antibiotic genotoxicity from a sporadic observational signal toward a tractable mechanistic field. The proposal by Kalghatgi and colleagues that bactericidal antibiotics induce mitochondrial dysfunction and oxidative damage in mammalian cells at clinically relevant doses [6] reframed the discussion from a collection of class-specific liabilities toward a possibly convergent off-target problem. That reframing remains debated. Subsequent work has both supported and qualified the original claims, and the magnitude of any contribution of ROS to mammalian antibiotic toxicity in vivo is not fully settled [19].

Three integrative frameworks now provide useful scaffolding: the adverse outcome pathway (AOP) concept, replication stress as a unifying intermediate node in chemical genotoxicity [20,21], and the recognition of ferroptosis as an iron- and lipid-peroxidation-driven cell-death program with downstream DNA-damaging consequences [22]. Each of these concepts originated outside antibiotic toxicology, and their application here remains a recent and incomplete project.

### 1.3. Direct Versus Indirect Genotoxicity

**Figure 1:**
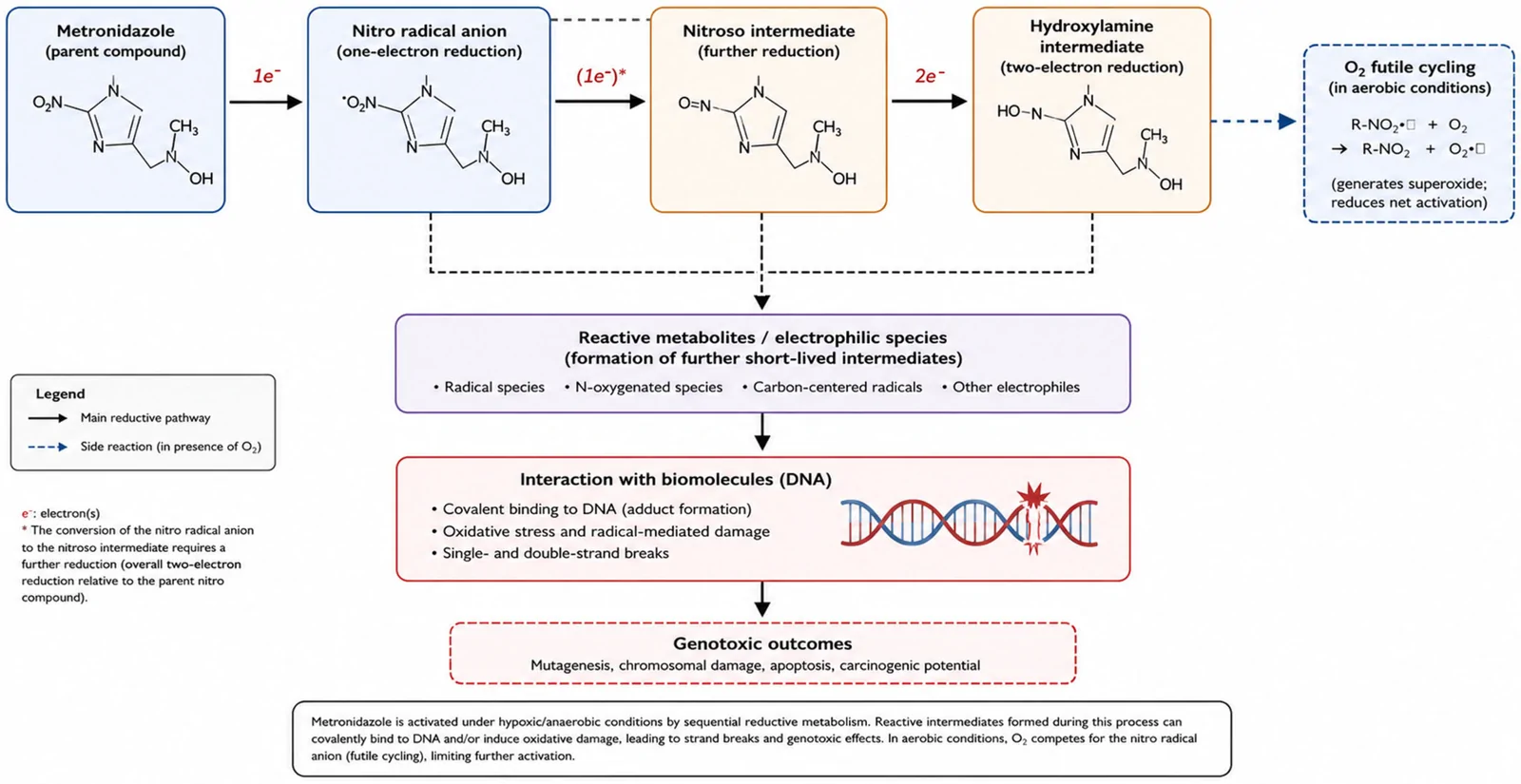
The bioreductive activation pathway of metronidazole and DNA adduct formation. Sequential one-electron reductions convert the parent nitro compound (R-NO_2_) through a nitro-radical anion intermediate to nitroso and hydroxylamine species that form covalent DNA adducts, primarily at guanine N-2 and C-8 positions. In aerobic conditions, futile cycling with molecular oxygen regenerates the parent compound and produces superoxide, limiting but not eliminating genotoxic activation in normoxic host tissues. Dashed arrows indicate sequential mechanistic steps from antibiotic–target interaction to the proximal genotoxic lesion.

The distinction between direct and indirect genotoxicity is foundational, though more porous in practice than is sometimes implied. Direct genotoxins interact covalently or sterically with DNA itself, or with enzymes that maintain genome integrity. Fluoroquinolones provide the clearest example: at certain intracellular concentrations they bind eukaryotic topoisomerase IIα and IIβ, generating trapped cleavage complexes that mature into protein-linked double-strand breaks upon collision with replication forks or transcribing RNA polymerase II [12,13,23]. Nitroimidazoles, metronidazole in particular, undergo one-electron bioreductive activation in hypoxic or microaerophilic conditions, yielding nitro-radical anions and covalent DNA adducts (Figure 1). This selectivity for anaerobes is operationally useful but biochemically incomplete in mammalian tissues with locally low oxygen tension, such as abscess cavities, tumor microenvironments, and segments of the intestinal mucosa [8,24,25].

Indirect genotoxicity, by contrast, is mediated through cellular stress responses. Oxidative stress, mitochondrial dysfunction, inflammation, and perturbation of replication and repair machinery are the principal effectors. Aminoglycosides and macrolides exemplify this mode. By binding mammalian mitoribosomes whose evolutionary origin is bacterial, they reduce synthesis of mtDNA-encoded electron-transport-chain subunits, destabilize respiratory complex assembly, and amplify mitochondrial reactive oxygen species [26,27,28]. β-Lactams and tetracyclines, often regarded as the least intrinsically genotoxic classes, can produce measurable signals in defined experimental contexts. In the β-lactam case, much of this signal is microbiome-mediated, propagating through bile-acid metabolism to colonocyte oxidative stress [29,30]. The boundary between direct and indirect mechanisms is porous: the fluoroquinolone inhibition of mitochondrial topoisomerase II is simultaneously direct mtDNA injury and an initiator of indirect ROS amplification [23,31].

### 1.4. Why a Mechanistic Synthesis Now

Clinical relevance is neither uniform nor trivial. Pediatric patients, especially neonates, have immature phase I and phase II metabolic systems, lower glutathione reserves, and developmentally distinct DNA-repair kinetics [32,33]. Children also experience the heaviest cumulative antibiotic exposure of any demographic group. Elderly patients accumulate genotoxic burden through polypharmacy, comorbidity, declining repair fidelity, and reduced renal clearance that prolongs tissue exposure to several genotoxic antibiotic classes [34]. Pregnant women constitute a third high-stakes population in which transplacental exposure intersects with embryonic and fetal cells that are proliferating rapidly and depend on faithful chromosome segregation. Oncology patients frequently receive antibiotic prophylaxis alongside cytotoxic chemotherapy or radiation; additive or synergistic genotoxic stress is supported by current evidence but rarely formally quantified.

Methodologically, the field has matured without becoming uniform. The CBMN-Cyt assay, comet assay, chromosomal aberration test, sister chromatid exchange assay, γH2AX foci assay, and TUNEL each capture a distinct biological dimension of genotoxic injury. Their concordance in antibiotic studies is variable, and inter-laboratory standardization remains uneven [16,18,35]. Oxidative and mitochondrial biomarker panels (8-hydroxy-2′-deoxyguanosine, malondialdehyde, glutathione redox ratio, mtDNA copy number, and mitochondrial membrane potential) add interpretive depth but are sensitive to preanalytical confounders [36,37]. Where human biomonitoring data exist, the cohorts are typically small, and adjustment for the genotoxic effects of the underlying infection is often inadequate. Pediatric and pregnancy biomonitoring, the populations of greatest translational interest, are critically underrepresented. The emerging methodological repertoire (multi-omics integration, microphysiological systems, single-cell genotoxicology, and AI-assisted hazard prediction) provides architecture capable of addressing these gaps, but most of these approaches have yet to be applied at scale to antibiotic genotoxicity.

### 1.5. Scope, Selection Criteria, and Structure

This review provides a mechanism-driven synthesis rather than a descriptive enumeration of studies. We organize evidence around molecular initiating events, key event cascades, and translational endpoints, broadly aligned with the AOP framework but without claiming AOP-formal status. The six prioritized classes (fluoroquinolones, nitroimidazoles, aminoglycosides, macrolides, β-lactams, and tetracyclines) were selected based on three criteria: clinical use frequency across pediatric and adult populations, depth of the available mechanistic literature, and representation of distinct off-target liabilities.

We have prioritized in vitro studies using human-derived cells (where these inform mechanism), in vivo studies in mammalian models, and human biomonitoring cohorts. Environmental exposure data are integrated within the One Health framing of Section 8 but are not the primary focus. By framing antibiotic genotoxicity as a measurable safety parameter rather than a residual concern, we argue that antimicrobial stewardship discussions should consider host genome integrity in vulnerable populations, without overstating the strength of currently available human evidence.

### 1.6. The Literature Search Strategy

This is a narrative, mechanism-focused review rather than a formal systematic review, though we have applied transparent search and selection criteria to support reproducibility. The literature was retrieved from PubMed, Scopus, and Web of Science between September and 25 December 2025, covering the publication window of 2010 through 2025, with selective inclusion of pre-2010 foundational studies where they were mechanistically indispensable (e.g., the original CBMN-Cyt protocol; m.1555A>G discovery). A small number of publications from early 2026 that appeared after the formal search closure were identified through citation alerts and author awareness during the revision process and were incorporated where they provided important mechanistic or methodological contributions not available in the original search results.

Core search terms combined antibiotic terminology (‘antibiotic’, ‘antimicrobial’, and class- or drug-specific terms, including ‘fluoroquinolone’, ‘ciprofloxacin’, ‘metronidazole’, ‘gentamicin’, ‘azithromycin’, ‘doxycycline’, ‘amoxicillin’, and ‘tetracycline’) with genotoxicity terminology (‘genotoxicity’, ‘DNA damage’, ‘cytogenetic’, ‘micronucleus’, ‘comet assay’, ‘chromosomal aberration’, ‘γH2AX’, ‘8-OHdG’, ‘oxidative DNA damage’, and ‘mitochondrial dysfunction’). MeSH terms were used where applicable in PubMed; equivalent controlled vocabulary was applied in Scopus and Web of Science.

Inclusion criteria were peer-reviewed publications in English; a mammalian or human study system; a mechanistic, cytogenetic, or biomonitoring focus; and sufficient methodological detail for critical appraisal. Exclusion criteria were a purely ecotoxicological scope (aquatic or plant systems without translational extrapolation); studies addressing antimicrobial mechanisms without host genome assessment; conference abstracts without full publication; publications in journals with questionable peer-review practices, identified using established checklists; and retracted articles. We prioritized recent mechanistic studies (2020–2026), cytogenetic studies with adequate technical reporting consistent with the MIRCA framework for the comet assay [35] and HUMN-project conventions for the micronucleus assay, and human biomonitoring studies regardless of cohort size. Citation tracking and recommendations from colleagues with domain expertise in genetic toxicology and cytogenetics supplemented database retrieval, particularly for emerging topics (microphysiological systems, single-cell genotoxicology, and ferroptosis). The reference list represents a curated subset rather than an exhaustive enumeration, and we have flagged areas where the available evidence is preliminary or of limited generalizability. As this review was designed as a narrative synthesis, quantitative records of the number of citations identified, screened, excluded, and included at each stage were not maintained during the search process. Consequently, a PRISMA-compliant flow diagram with numerical data cannot be provided. This is a methodological limitation; however, the narrative approach was considered appropriate given the heterogeneity of study designs, endpoints, and model systems covered, which would not lend themselves to the standardized eligibility framework of a systematic review.

## 2. Antibiotic Classes and Mechanisms of Action

**Figure 2:**
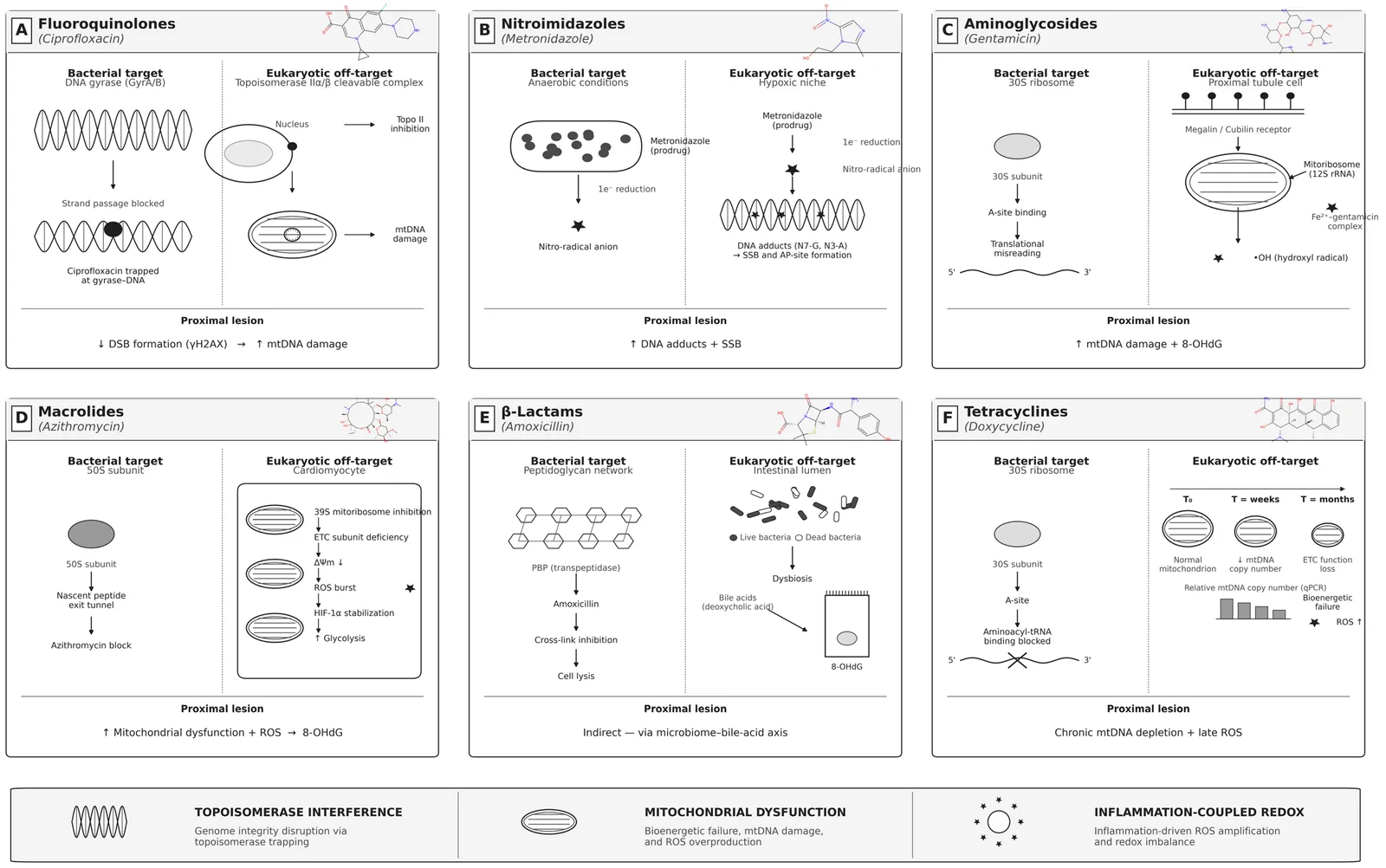
A mechanistic overview of antibiotic-induced genotoxicity for the six prioritized classes. Each panel presents the bacterial target, eukaryotic off-target, key molecular pathway, and proximal genotoxic lesion. Three convergent damage axes are identified: topoisomerase interference (fluoroquinolones), mitochondrial dysfunction (aminoglycosides, macrolides, and tetracyclines), and inflammation-coupled redox imbalance. ↑ = increase; ↓ = decrease in the indicated parameter. × = inhibition or loss of function.

**Figure 3:**
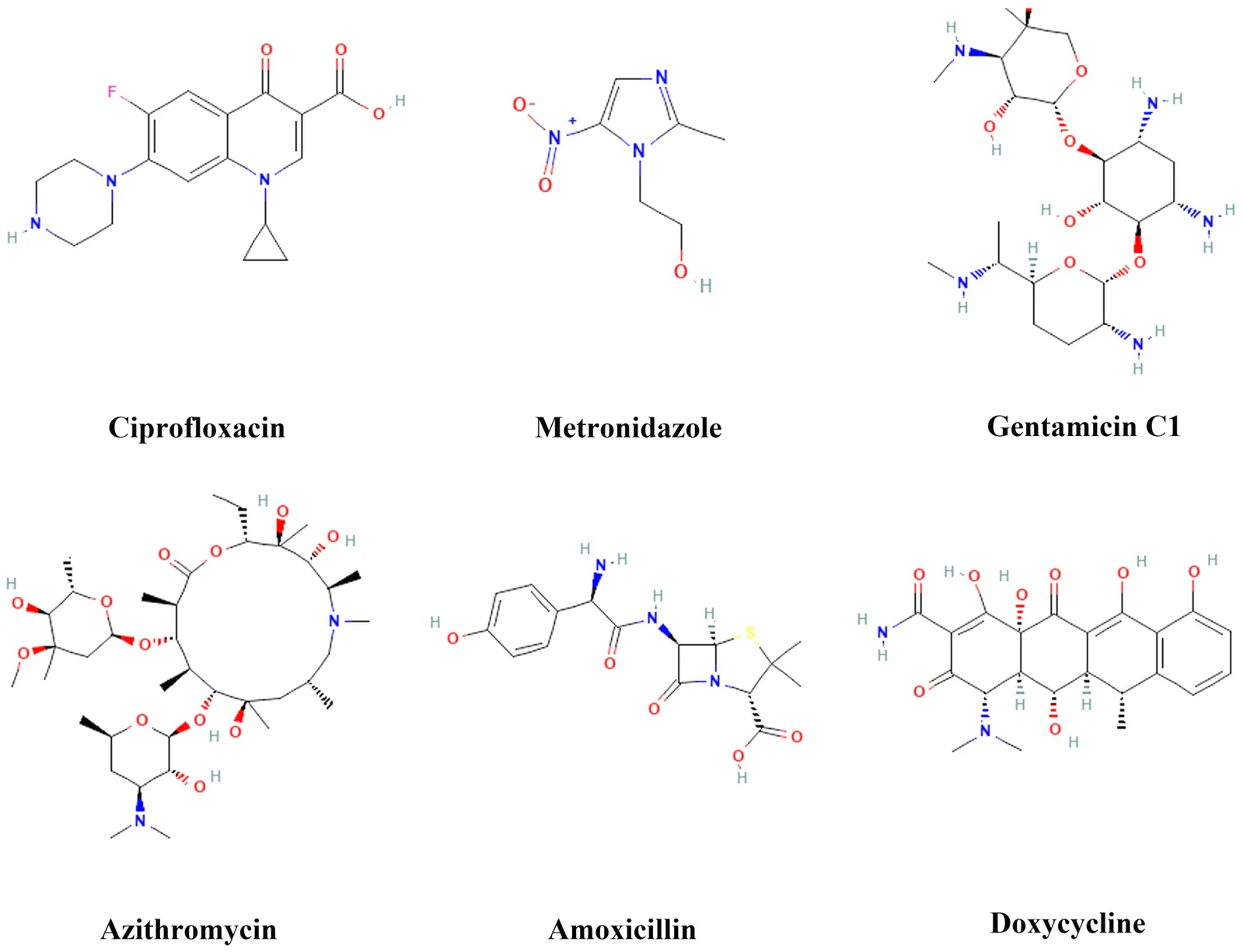
Chemical structures of six representative antibiotics discussed in this review: ciprofloxacin (fluoroquinolone), metronidazole (nitroimidazole), gentamicin C1 (aminoglycoside), azithromycin (macrolide/azalide), amoxicillin (β-lactam/penicillin), and doxycycline (tetracycline).

A class’s genotoxic profile is set by three things: the molecular identity of its bacterial target, whether mammalian cells encode a structurally similar counterpart, and the pharmacokinetic envelope across human tissues. The first two are biochemical features of the molecule; the third decides whether mechanisms demonstrated in vitro have any chance to operate clinically. Section 2 walks through six prioritized classes within this frame (Table 1; Figure 2). A central evidence summary of the key studies discussed in this review, organized by antibiotic class, experimental model, measured genotoxic endpoint, and clinical relevance, is provided in Table 2. The chemical structures of representative agents from these six classes are presented in Figure 3. Where the evidence supports a mechanism at clinically achievable tissue concentrations, we say so; where the in vitro signal is real but only emerges at supratherapeutic doses, we say that; and where claims rest on extrapolation, we mark them as such, rather than allowing hedged phrasing to imply more than the data support.

### 2.1. Fluoroquinolones

**Figure 4:**
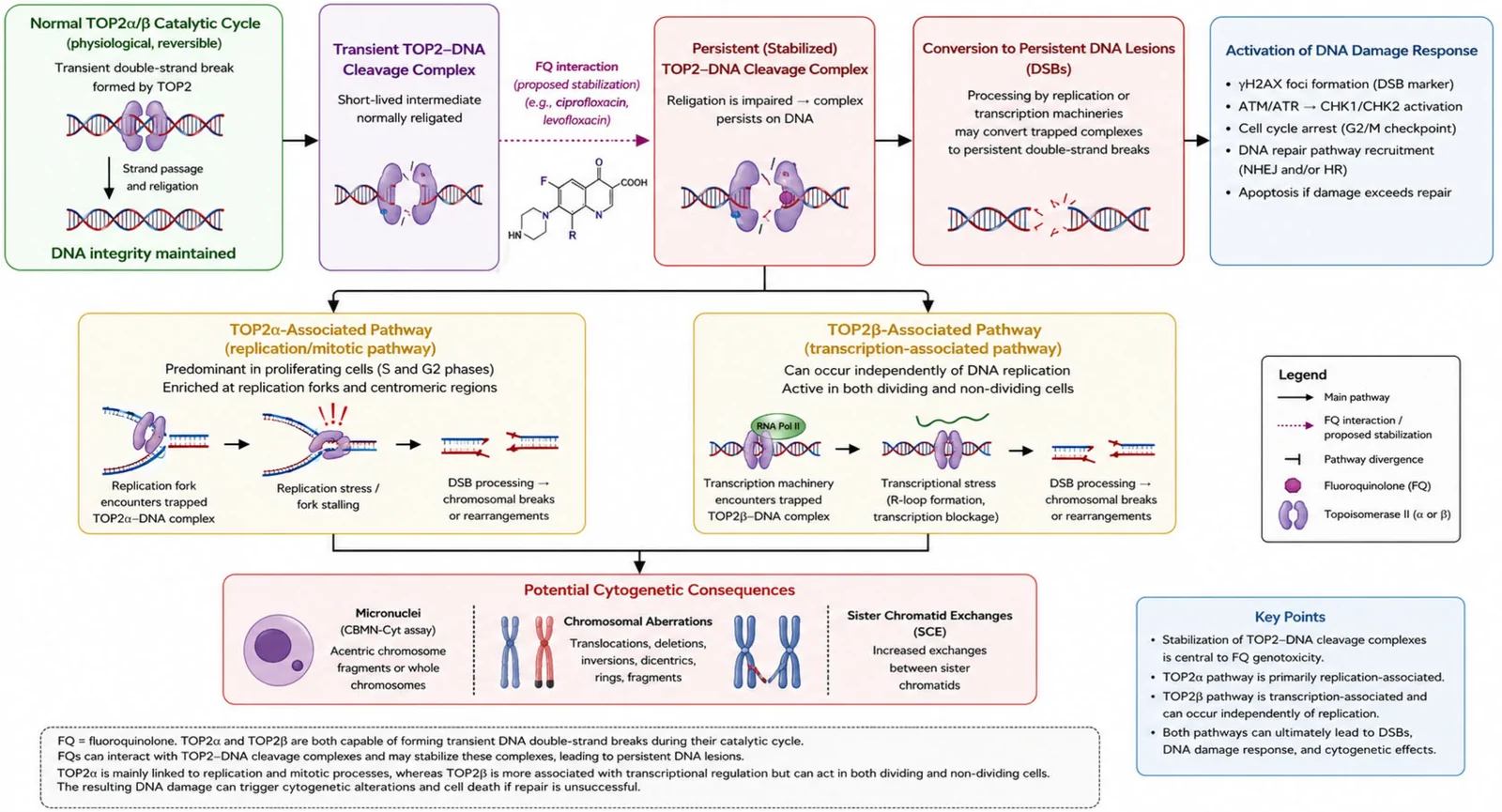
Fluoroquinolone–topoisomerase II cleavage complex mechanism and downstream genotoxic consequences. Fluoroquinolones stabilize the covalent Topo II–DNA cleavage complex, preventing religation. Collision with replication forks converts trapped complexes into irreversible double-strand breaks (DSBs), triggering DNA damage response and, if mis-repaired, cytogenetic damage detectable as micronuclei, chromosomal aberrations, or sister chromatid exchanges. The Topo IIβ-mediated pathway generates transcription-associated DSBs independent of replication.

Ciprofloxacin, levofloxacin, and moxifloxacin trap bacterial DNA gyrase and topoisomerase IV in cleavage complexes [41,42]; the mammalian counterparts they cross-react with are Topo IIα and Topo IIβ. That cross-reactivity is biochemically real. Lawrence et al. [23] documented concentration-dependent mtDNA cleavage in HepG2 cells from 50 to 200 μM ciprofloxacin; Hangas et al. [12] showed mtDNA replication impairment in HeLa at similar exposures; and Smart and Lynch [38] reported γH2AX foci induction in TK6 cells from 25 μM. The sequential steps from fluoroquinolone binding through cleavage-complex stabilization to irreversible DSB formation and downstream cytogenetic consequences are illustrated in Figure 4. The interpretively relevant question is how these concentrations relate to clinical pharmacology. Plasma Cmax at standard 500 mg oral ciprofloxacin dosing is reported in the range of approximately 2–4 μg/mL (≈76–12 μM) in pharmacokinetic studies of healthy volunteers; at the lower 200 mg dose studied by Drusano et al. [43], oral Cmax averaged 1.2 μg/mL (≈3.6 μM). This gives a nominal 5- to 40-fold gap to the in vitro genotoxic threshold, depending on dose. The gap narrows substantially in tissues that accumulate fluoroquinolones: tendon, articular cartilage, renal cortex, and certain ocular structures are reported to concentrate ciprofloxacin to approximately 3- to 5-fold above concurrent plasma concentrations based on tissue homogenate measurements, potentially bringing local total-drug exposure into the 15–50 μM range under repeated dosing; however, these values represent total tissue concentrations and may overestimate free intracellular drug available for interaction with nuclear targets within range of the in vitro thresholds at which Topo II poisoning becomes detectable. Trapped Topo II–DNA covalent complexes are normally transient intermediates of strand passage; in their drug-stabilized form they cannot religate, and collision with replication forks or transcribing RNA polymerase II produces protein-linked double-strand breaks [13,44,45]. This places fluoroquinolones in the same broad pharmacological category as established Topo II poisons, such as etoposide, but the cross-class comparison is misleading at the dose level: etoposide is administered to achieve cytotoxic Topo II inhibition, while fluoroquinolones are administered at exposures where the effect is sub-cytotoxic and accumulative.

Three additional mechanisms contribute and are worth separating rather than bundling. First, fluoroquinolones inhibit mitochondrial topoisomerase II and impair mtDNA replication; the threshold is lower than for nuclear Topo II because mtTopo II lacks the redundancy of the nuclear compartment [23,31]. Second, intracellular divalent-cation chelation perturbs the labile iron pool and seeds Fenton-type hydroxyl-radical chemistry [46]; the magnitude is concentration-dependent and is not well-characterized at clinical tissue exposures. Third, partial uncoupling of oxidative phosphorylation and elevated mitochondrial permeability transition have been reported across several systems with ROS spillover to the cytosol, but the experimental designs vary widely, and the effect sizes are difficult to compare across reports. The clinical adverse-event signature does not map cleanly onto any single mechanism. Tendinopathy and aortopathy have the strongest population-level epidemiologic support [47,48] and are most plausibly explained by extracellular matrix disruption combined with oxidative stress in collagen-rich tissues. Peripheral neuropathy implicates mitochondrial dysfunction in Schwann cells. The persistent multisymptom disability syndrome characterized by Golomb et al. [49] remains the least understood phenotype and likely reflects a composite of mitochondrial and oxidative injury in cells with limited regenerative capacity. Tissue accumulation patterns (highest in tendon, articular cartilage, renal cortex, and ocular structures) parallel the clinical adverse-event topography, an observation that constrains hypotheses about the underlying cellular biology without proving any one of them.

### 2.2. Nitroimidazoles

Metronidazole and its analogs (tinidazole, ornidazole, and secnidazole) are prodrugs activated by enzymatic one-electron reduction of the nitro group. The nitro-radical anion abstracts hydrogen atoms from DNA, forms covalent adducts at guanine N7 and adenine N3 positions, and propagates single- and double-strand breaks through abasic-site intermediates [8,24,50]. Selectivity for anaerobic pathogens derives from rapid reoxidation of the radical in oxygen-replete tissue, but the selectivity is not absolute: hypoxic niches sustain reductive activation in host cells, with measurable consequences in tumor microenvironments, abscess cavities, and segments of the intestinal mucosa [51]. The cytogenetic literature on this class is genuinely conflicted. Chronic high-dose administration produces reproducible CBMN, comet, and SCE signals in lymphocyte studies [9,39,40]; the standard 7- to 10-day therapeutic courses typically used for anaerobic infections give weaker or equivocal results; and the well-controlled dog RCT by Peterson et al. [52] reported no in vivo genotoxicity at therapeutic doses despite clear in vitro positivity for the same compound. IARC classified metronidazole as possibly carcinogenic to humans (Group 2B) in 1987 on the basis of rodent carcinogenicity and mechanistic plausibility [53]; the classification has not been revisited, despite four decades of accumulating pharmacoepidemiology that has not consistently identified excess cancer in exposed populations [54]. The discrepancy between robust preclinical signals and weak clinical signals is the central interpretive problem with this class and is a useful case study in why mechanistic genotoxicity does not automatically translate into human carcinogenic risk.

### 2.3. Aminoglycosides

Gentamicin, amikacin, tobramycin, and streptomycin bind the bacterial 30S ribosomal subunit at the A-site decoding center, inducing translational misreading [55]. The mammalian mitoribosome is evolutionarily related and binds aminoglycosides with measurable affinity. That affinity is amplified by mtDNA polymorphisms—principally m.1555A>G and m.1494C>T in 12S rRNA—that underlie inherited aminoglycoside-induced deafness [10,56]. This is one of the few examples in the antibiotic–genotoxicity field where a specific molecular mechanism (mitoribosome binding at the 12S decoding site) maps directly onto a specific clinical syndrome (sensorineural hearing loss) with strong genotype–phenotype data and a defined penetrance gradient. Beyond mitoribosomal inhibition, aminoglycosides form iron complexes that catalyze Fenton-type hydroxyl-radical generation at the inner mitochondrial and plasma membrane interfaces [57,58]. Tissue distribution amplifies the problem rather than mitigating it. Renal proximal tubular cells take up aminoglycosides via megalin/cubilin-mediated endocytosis, reaching intracellular concentrations 30- to 50-fold above plasma; cochlear sensory hair cells retain them through mechanotransduction channels [59,60]. Plasma Cmax at standard 5–7 mg/kg gentamicin dosing reaches 5–10 μM, so the estimated renal cortical concentration during a 7-day course, calculated from tissue accumulation ratios rather than direct intracellular measurement, may exceed 250 μM—far above the threshold for measurable in vitro mitochondrial dysfunction. This is the rare instance in this review where pharmacokinetics, biochemistry, and clinical phenotype align without interpretive strain: aminoglycoside nephrotoxicity and ototoxicity are not merely clinically observed but mechanistically expected. The trajectory from accumulation to injury proceeds through mitochondrial dysfunction, ROS overproduction, mtDNA damage, ΔΨm collapse, and apoptotic or ferroptotic cell death [58]; the precise sequencing of steps continues to be refined but the broad pathway is well-supported.

### 2.4. Macrolides

Azithromycin, clarithromycin, and erythromycin bind the bacterial 50S subunit at the nascent peptide exit tunnel and block translational elongation [61]. They engage mammalian mitoribosomes (39S large subunit) with consequences for synthesis of mtDNA-encoded ETC components and assembly of complexes I and IV [62,63]. The most-cited mechanistic study is from Jiang et al. [63], which reported that azithromycin in human mammary epithelial cells and primary fibroblasts collapses ΔΨm within hours, drives mitochondrial superoxide bursts, elevates urinary 8-OHdG, and triggers a Warburg-like shift to aerobic glycolysis with HIF-1α stabilization. The findings are robust at the cellular level but constrained: the cell types were selected for high mitochondrial activity, exposures were generally at or above peak plasma levels, and no parallel measurements were performed in cardiomyocytes—the very cell type most relevant to clinical macrolide cardiotoxicity. Whether the same mitochondrial signature occurs in cardiomyocytes at clinical exposure remains the central unresolved question, particularly because azithromycin’s exceptionally long tissue half-life (60–80 h) and 10-to-100-fold tissue-to-plasma concentration ratio [64], measured primarily by HPLC in tissue homogenates. Serum protein binding of azithromycin is concentration-dependent, declining from approximately 50% at 0.02 mg/L to 12% at 0.5 mg/L [64], so that unbound drug fractions are relatively high at clinically relevant concentrations. Tissue homogenate values represent the total drug and may overestimate free intracellular concentrations available for interaction with host-cell DNA, making local exposure substantially higher than plasma values would suggest. The clinical correlate is the QT prolongation and arrhythmia signal that prompted FDA caution and the cardiovascular mortality association reported by Ray et al. [65]; mitochondrial dysfunction provides a plausible mechanistic substrate, but no study has directly demonstrated the link between the in vitro mitochondrial phenotype and clinical arrhythmogenesis. Macrolides additionally exert immunomodulatory effects (NF-κB suppression, IL-6/IL-8 downregulation, and neutrophil functional modulation) that can be protective in acute inflammation-driven contexts and potentially deleterious under chronic prophylactic dosing where impaired clearance of damaged cells could allow genotoxic lesions to accumulate [66].

### 2.5. β-Lactams

Penicillins, cephalosporins, and carbapenems inhibit penicillin-binding proteins (PBPs) involved in bacterial peptidoglycan crosslinking and have no orthologous target in eukaryotic cells [67]. They are accordingly among the least intrinsically genotoxic antibiotic classes. The great majority of in vitro cytogenetic and comet assay studies at therapeutic concentrations return negative or marginal results. Idiosyncratic ROS generation has been reported at high concentrations [6], and inhibition of the mitochondrial carnitine/acylcarnitine transporter has been documented for some β-lactams, potentially perturbing fatty-acid β-oxidation and indirectly elevating ROS [68]. The more biologically significant indirect mechanism, however, appears to be microbiome disruption: β-lactam-induced depletion of commensal anaerobes, expansion of facultative species, and altered bile-acid and short-chain-fatty-acid metabolism can elevate colonocyte genotoxic stress through secondary bile acids, most prominently deoxycholic acid, which have been shown to generate ROS and 8-OHdG in intestinal epithelium [30,69]. Perinatal and early-infancy β-lactam exposure has been associated with differential DNA methylation at imprinted loci, suggesting that even classes with minimal direct genotoxic liability may produce epigenetically consequential indirect effects during developmental windows [70,71]. These associations, however, remain associative rather than causal, and confounding by indication is difficult to exclude in observational cohorts.

### 2.6. Tetracyclines

Doxycycline, minocycline, and tetracycline bind the 30S ribosomal subunit and block aminoacyl-tRNA delivery [72]. They also inhibit mitochondrial translation in a dose- and time-dependent manner, with measurable depletion of mtDNA-encoded ETC subunits after prolonged exposure, and they inhibit matrix metalloproteinases (the latter providing the mechanistic basis for several off-label uses including in acne, rosacea, and periodontal disease) [73,74]. Mitochondrial translation inhibition is reversible at standard short-course therapeutic exposures but becomes mechanistically consequential during the months-to-years dosing regimens common in dermatologic and rheumatologic indications. Under prolonged exposure, measurable reductions in mtDNA copy number, bioenergetic capacity, and respiratory complex activity have been documented, with secondary elevations of ROS and oxidative DNA damage [74]. The genotoxic profile of tetracyclines, therefore, differs qualitatively from that of fluoroquinolones or nitroimidazoles: chronic, accumulative, and mitochondrially mediated rather than acute and direct. Whether this profile translates into measurable long-term human cytogenetic or carcinogenic risk under typical dermatologic regimens is unknown.

### 2.7. Beyond the Six Prioritized Classes: Glycopeptides and Glycylcyclines

Although the six prioritized classes account for the majority of clinical antibiotic prescriptions and the bulk of the mechanistic genotoxicity literature, two additional structurally distinct classes warrant brief mention. Glycopeptides (vancomycin and teicoplanin) target bacterial cell-wall biosynthesis by binding D-Ala-D-Ala termini of peptidoglycan precursors. There is no obvious eukaryotic counterpart. Despite this apparent target selectivity, recent combined experimental and in silico evaluation has documented measurable cytotoxic and genotoxic signals for teicoplanin in mammalian cells, with Vega Hub and Toxtree computational predictions reinforcing the experimental findings. This QSAR-plus-bench framework illustrates the integrative approach adopted in regulatory toxicology, particularly for less-studied classes [75]. Glycylcyclines, the structurally modified tetracycline derivatives exemplified by tigecycline, share the mitochondrial-translation liability of their parent class. A recent in vivo study using the alkaline comet assay in bladder tissue of rats receiving intravesical tigecycline reported dose-independent genotoxic damage at both therapeutic and sub-therapeutic doses, despite robust microbiological efficacy against extensively drug-resistant Acinetobacter baumannii [76]. The relevance of these animal findings to human bladder tissue under clinical intravesical regimens is not established. The broader implication is that the mechanistic axes developed throughout this review, particularly mitochondrial perturbation and oxidative stress, likely operate across a wider antibiotic landscape than the six prioritized classes alone, and that integrated cytogenetic-plus-computational hazard assessment may be especially useful where conventional in vivo data are sparse.

### 2.8. Three Convergent Off-Target Axes

Three mechanisms reappear across the classes covered above (Table 3). Eukaryotic topoisomerase II interference is largely a fluoroquinolone phenomenon and produces direct DSBs through trapped cleavage complexes. Mitochondrial perturbation is shared more broadly—aminoglycosides and macrolides through mitoribosomal inhibition, tetracyclines through chronic translation suppression, and fluoroquinolones through mitochondrial Topo II—and produces secondary ROS, mtDNA damage, and bioenergetic failure. Inflammation-coupled oxidative stress operates indirectly through microbiome dysbiosis (β-lactams) or directly through cellular ROS amplification, although the strength of the latter has been disputed since Liu and Imlay [19] demonstrated that bacterial cell death can occur without ROS involvement, weakening the original Kohanski hypothesis as a universal explanation. These mechanisms are not independent. Mitochondrial dysfunction increases ROS, which exacerbates topoisomerase-mediated damage; cytokines from damaged cells amplify ROS further; and oxidatively modified repair proteins close a feed-forward loop in which initial lesions become progressively harder to resolve. The framework organizes the evidence but should not be over-extended: most supporting data are preclinical, the quantitative contributions at clinical exposures are uncharacterized, and a PBPK-informed dose-response analysis across classes and tissues has not been performed. Strength of evidence varies sharply by class and endpoint (Table 4); treating “antibiotic genotoxicity” as a homogeneous category obscures the distinctions that actually matter for clinical risk stratification. (i) Fluoroquinolones—strong mechanistic and moderate human evidence at sites of tissue accumulation. (ii) Nitroimidazoles—strong in vitro evidence but a persistently weak clinical signal. (iii) Aminoglycosides—coherent mechanism-to-phenotype mapping at the renal-cortical and cochlear sites of accumulation. (iv) Macrolides—robust in vitro mitochondrial findings without a direct clinical mechanistic link. (v) β-Lactams—minimal direct genotoxicity, indirect microbiome-mediated effects under developmental exposure. (vi) Tetracyclines—chronic-exposure, accumulative mitochondrial liability of unclear human consequence.

## 3. Molecular Mechanisms of Antibiotic-Induced Genotoxicity

Having outlined the structural and pharmacological heterogeneity of antibiotic classes, we now examine the molecular mechanisms through which these structurally diverse agents converge on a shared spectrum of DNA lesions. The available evidence spans the full hierarchy of genotoxic events: initial physicochemical interactions with DNA, perturbation of replication and transcription, activation of DNA damage response (DDR) signaling, and engagement of repair, apoptosis, or senescence programs. Throughout this section, we distinguish mechanisms that are firmly established from those that remain supported by current evidence but rest on more limited evidence.

### 3.1. Direct DNA Interaction: Adducts, Intercalation, and Topoisomerase Poisoning

Among the classes considered here, fluoroquinolones and nitroimidazoles have the most clearly documented capacity to interact with eukaryotic DNA directly or through covalent enzyme intermediates. Fluoroquinolone-induced Topo II cleavage complexes are stabilized by intercalation of the drug between cleaved DNA ends and the enzyme dimer interface, blocking ATP-dependent religation [44,45]. When subsequently encountered by an advancing replisome or by elongating RNA polymerase II, these cleavage complexes are processed by tyrosyl-DNA phosphodiesterase 2 (TDP2) and the MRE11–RAD50–NBS1 (MRN) complex into frank DSBs [77]. Topo IIβ-mediated breaks in terminally differentiated tissues (neurons, cardiomyocytes, and tendinocytes) have been implicated in the chromosomal translocation events that characterize secondary leukemias following Topo II–poison chemotherapy. The same mechanism is biologically reasonable for fluoroquinolone exposure, though the quantitative magnitude appears substantially smaller [13,78].

Nitroimidazole-derived adducts are recognized predominantly by the base excision repair (BER) machinery [79]. The nitro-radical anion abstracts hydrogen atoms from the sugar–phosphate backbone or from heterocyclic bases, forming covalent guanine and adenine adducts and abasic sites that mature into single-strand breaks during replication. When BER capacity is overwhelmed or when adducts cluster, replication-fork collapse can generate secondary DSBs. These two mechanisms, Topo II–linked DSBs and reductively or oxidatively modified bases, produce biochemically distinct lesions whose cytogenetic signatures differ accordingly. The former is more strongly associated with chromosomal translocations; the latter with point mutations and acentric-fragment micronuclei.

### 3.2. Replication Stress as a Unifying Intermediate Node

Replication stress is recognized as a unifying intermediate node in chemical genotoxicity broadly, and antibiotic-induced genotoxicity may fit this framework [20,21]. Antibiotic-induced lesions (direct adducts, oxidized bases, or topological obstacles) can slow or stall replication forks, deplete the dNTP pool, and uncouple the replicative helicase from the polymerase complex, generating long stretches of single-stranded DNA bound by replication protein A (RPA) [80]. RPA-coated ssDNA recruits the ATR-ATRIP complex and activates ATR kinase signaling, the canonical sensor of replication stress. Persistent stalling that fails to resolve through fork restart, dormant origin firing, or controlled fork reversal can culminate in fork collapse and DSB formation [21,81]. Several antibiotics, notably ciprofloxacin and high-dose metronidazole, have been shown to elevate markers of replication stress in mammalian cell lines: RPA32 hyperphosphorylation, CHK1 activation, and slowed fork progression measured by DNA fiber assays [23,82]. These data come from immortalized cell lines under defined experimental conditions; their relevance to slowly dividing tissues in vivo is less clear.

### 3.3. DNA Damage Response Activation

Cellular response to antibiotic-induced lesions follows the canonical PIK-related kinase hierarchy. ATM responds principally to DSBs detected by the MRN complex. ATR responds to single-stranded DNA and stalled forks detected by RPA-ATRIP. DNA-PKcs responds to DSBs destined for non-homologous end joining (NHEJ), detected by the Ku70/80 heterodimer [83,84]. All three kinases phosphorylate histone H2AX on Ser139 to generate γH2AX, which spreads in megabase-scale chromatin domains around each break and serves as a platform for MDC1, 53BP1, BRCA1, and downstream mediator recruitment [14,15,85]. γH2AX foci accumulate within minutes of clinically relevant ciprofloxacin or metronidazole exposure in vitro and constitute one of the most operationally tractable molecular biomarkers of antibiotic-induced DSBs available.

Downstream signaling diverges along two principal branches. ATM activates the CHK2–p53–p21 axis, predominantly driving G1/S checkpoint enforcement and apoptotic induction in cells with high lesion burden. ATR activates CHK1, which inactivates the CDC25A/B/C phosphatases and enforces G2/M arrest [84,86]. BRCA1 and 53BP1 antagonistically direct repair pathway choice between homologous recombination and NHEJ. The kinetics of this choice can be perturbed in cells with antibiotic-induced replication stress, biasing repair toward more error-prone alternatives. The integrated DDR network functions as both a damage-resolution machinery and a quality-control filter: cells whose damage load exceeds the threshold for faithful repair are diverted into apoptosis or senescence, preventing transmission of mutational burden to daughter cells.

### 3.4. Repair Pathway Failure Modes

When canonical repair pathways are overwhelmed or unavailable, three failure modes contribute to chromosomal instability. Microhomology-mediated end joining (MMEJ), driven by DNA polymerase θ (POLQ), generates short deletions and chromosomal translocations characterized by microhomology at breakpoints. MMEJ has been identified as the principal source of complex genomic rearrangements following Topo II poisoning [87,88]. Translesion synthesis (TLS) by Y-family polymerases (Pol η, ι, κ, and REV1) bypasses unrepaired lesions at the cost of introducing point mutations [89]. Mitotic entry with unrepaired DSBs produces lagging chromosomes, anaphase bridges (the cytogenetic correlate of which is the nucleoplasmic bridge), and acentric fragments visualized as micronuclei; all are scored in the CBMN-Cyt assay [16,90]. Together, these failure modes generate the chromosomal instability phenotype that, in oncologic contexts, is a hallmark of malignant transformation. The contribution of antibiotic-induced lesions to this phenotype outside oncology contexts is supported by current evidence but not formally established.

### 3.5. Apoptosis and Senescence

Cells unable to resolve genotoxic stress engage p53 and pursue one of two principal terminal fates: intrinsic apoptosis or replicative senescence. Intrinsic apoptosis proceeds through BAX/BAK oligomerization at the outer mitochondrial membrane, mitochondrial outer-membrane permeabilization, cytochrome c release, Apaf-1 apoptosome assembly, and caspase-9/-3 activation [91]. Antibiotics with strong mitochondrial liabilities (aminoglycosides, macrolides, and fluoroquinolones) appear to preferentially engage this pathway, because their off-target effects converge on the organelle that orchestrates apoptotic execution [58,63]. Replicative senescence is favored under chronic low-dose exposure and is characterized by p21 induction, persistent γH2AX foci, and the senescence-associated secretory phenotype (SASP), through which senescent cells secrete IL-6, IL-8, TGF-β, and matrix metalloproteinases. The SASP can in turn propagate paracrine genotoxicity to neighboring tissue [92]. Both terminal fates have secondary consequences. Apoptosis eliminates damaged cells but mandates compensatory replication that itself can elevate mutational risk in proliferative tissues. Senescence retains damaged cells whose secretome maintains a chronic genotoxic microenvironment.

### 3.6. Cell-Cycle Checkpoint Dysregulation

Repeated antibiotic-induced activation of the G1/S and G2/M checkpoints, mediated through p53–p21 and CHK1/CHK2–CDC25, can contribute to a state of chronic proliferative slowdown. Under prolonged exposure, this same pressure may, paradoxically, select for checkpoint-attenuated clones, a phenomenon documented in long-term cell culture and inferred in chronically exposed tissues [93]. This selection pressure is potentially relevant to the carcinogenic-risk discussion in Section 8. Chronic genotoxic stress favors emergence of cells with compromised tumor-suppressor function (most notably p53), and the logic underlying t-AML risk associated with Topo II–poison chemotherapy is, in principle, though at substantially lower magnitude, applicable to chronic fluoroquinolone exposure. The quantitative gap between chemotherapeutic and therapeutic-antibiotic genotoxic burden is several orders of magnitude, and human epidemiologic data have not consistently identified excess hematological malignancy risk in fluoroquinolone-exposed populations. The mechanistic continuity exists; the clinical translation is uncertain.

## 4. Oxidative Stress and Mitochondrial Dysfunction

DNA damage responses are activated downstream of two principal lesion sources: direct interactions between antibiotics and DNA-processing enzymes, and indirect oxidative damage propagated through mitochondrial perturbation. This section addresses the second. The role of oxidative stress and mitochondrial dysfunction in antibiotic-induced genotoxicity has been one of the most actively debated topics in the field over the past fifteen years; the relative contribution of ROS to mammalian antibiotic toxicity in vivo is not settled, and the original Kohanski hypothesis [7] of bactericidal-ROS killing has been substantially complicated by Liu and Imlay’s demonstration [19] that bacterial death can occur without measurable ROS. The question for mammalian biology is narrower and more tractable: which antibiotic classes generate ROS in mammalian cells at clinically relevant exposures, in which cell types, and through which specific mitochondrial entry points?

### 4.1. Antibiotic-Driven ROS Generation

The hypothesis that bactericidal antibiotics kill bacteria in part through ROS-mediated effects, proposed by Kohanski and colleagues and extended by Kalghatgi and colleagues to mammalian cells, has been mechanistically influential but is not universally accepted [6,7,94]. Some subsequent studies have failed to reproduce the central claim, particularly under conditions where bacterial ROS were measured with alternative probes or where antioxidant supplementation was tested for protective effect. The currently most defensible position is that bactericidal antibiotics contribute to mammalian ROS under at least some experimental conditions, and that this contribution may be biologically meaningful in mitochondria-rich tissues, but that ROS is not the sole or always the dominant pathway of mammalian antibiotic toxicity.

With that caveat in mind, fluoroquinolones, aminoglycosides, and tetracyclines have all been reported to elevate mitochondrial superoxide production in mammalian cells using MitoSOX fluorescence, EPR spin-trapping, and mitoB chemical probes [6,58,63]. Superoxide generated within the mitochondrial matrix is dismutated by Mn-SOD to hydrogen peroxide, which diffuses into the cytosol and can be converted to hydroxyl radical via Fenton chemistry in the presence of labile iron. Aminoglycosides and fluoroquinolones, both of which form direct complexes with iron, are particularly relevant to this pathway [46,58]. Hydroxyl radicals are short-lived but highly reactive: they damage virtually any biomolecule within nanometers of their site of generation, including DNA bases (yielding 8-OHdG, FapyGua, and related products), polyunsaturated fatty acids (initiating lipid peroxidation), and proteins (yielding carbonylated derivatives).

### 4.2. Class-Specific Mitochondrial Liabilities

**Figure 5:**
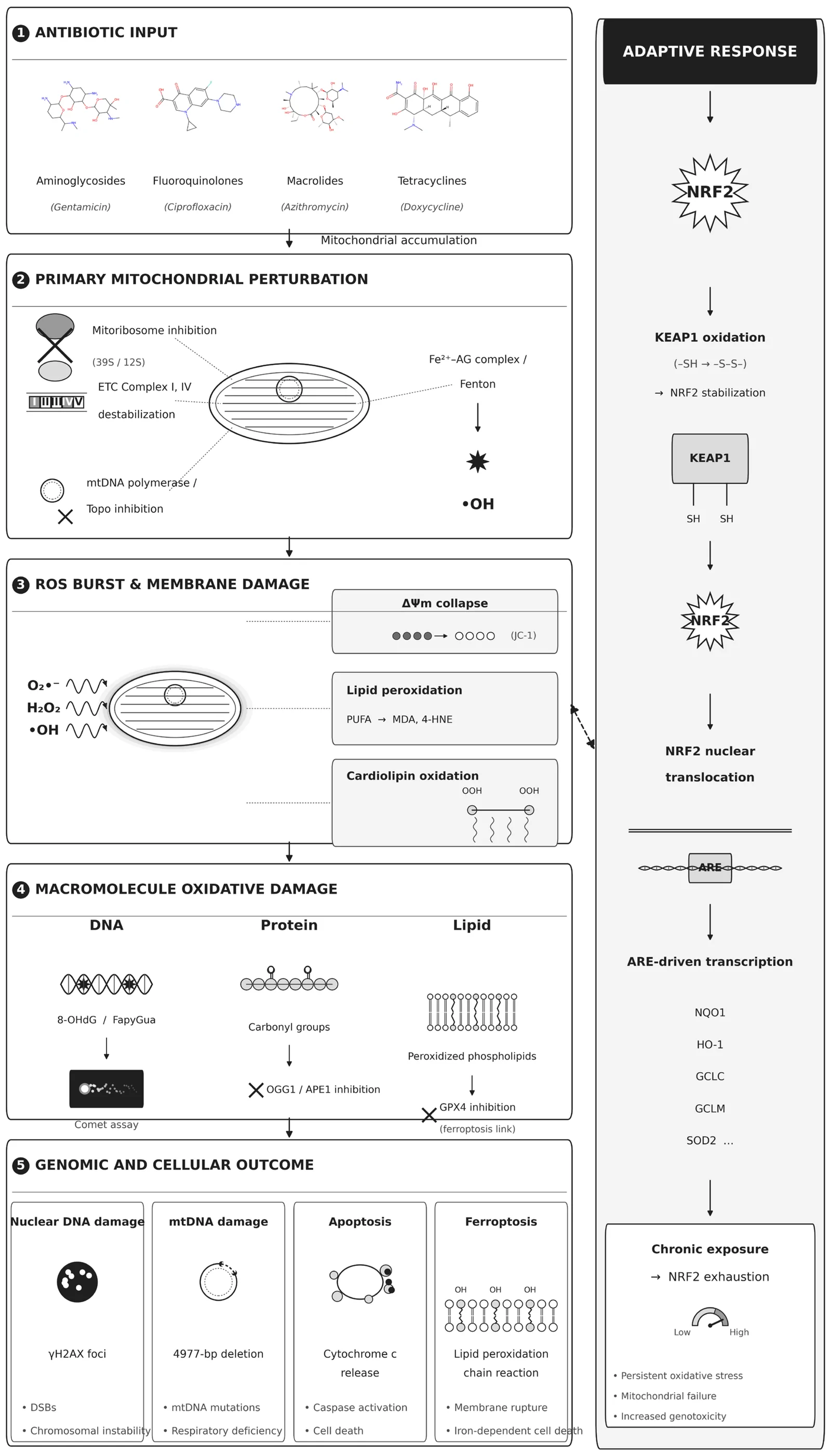
The convergent mitochondrial damage cascade triggered by the mechanistically distinct antibiotic classes. The pathway proceeds from the antibiotic input and mitochondrial accumulation through primary mitochondrial perturbation, ROS burst and membrane damage, and macromolecule oxidative damage to genomic and cellular outcomes. The NRF2-KEAP1 adaptive response pathway is shown in parallel.

In evolutionary terms, the mammalian mitochondrion is the most bacterial-like compartment of the eukaryotic cell. This origin underlies a recurring pattern in antibiotic toxicology: agents engineered to disrupt bacterial ribosomes, gyrases, or membranes can injure mitochondria through preserved structural homology [26,95]. Aminoglycosides and macrolides act directly on mitoribosomes (the 12S rRNA-containing 28S small subunit and the 39S large subunit, respectively), impairing translation of the thirteen mtDNA-encoded ETC subunits and destabilizing complex I and IV assembly [27,62]. Linezolid, an oxazolidinone outside the six prioritized classes but mechanistically informative, provides the clearest clinical demonstration of mitochondrial-translation toxicity: prolonged linezolid administration produces dose-dependent lactic acidosis, peripheral and optic neuropathy, and bone-marrow suppression, all attributable to mitochondrial translation inhibition [96]. Tetracyclines reduce mtDNA copy number and respiratory capacity over prolonged exposure [74]. Fluoroquinolones uncouple oxidative phosphorylation, elevate mitochondrial permeability transition pore opening, and inhibit mtDNA topoisomerase II, contributing to mitochondrial genome instability [23,47]. The convergent mitochondrial damage cascade triggered by these mechanistically distinct classes, and its downstream genotoxic consequences, is summarized in Figure 5.

The consequences of mitochondrial dysfunction extend beyond bioenergetic failure. Damaged mitochondria release mtDNA fragments into the cytosol, where they activate the cGAS-STING DNA-sensing pathway and trigger type I interferon responses that amplify inflammation [97,98]. They also lower the cellular threshold for intrinsic apoptosis by sensitizing the outer membrane to BAX/BAK oligomerization. The mitochondrion is, therefore, both a primary target of mitoribosome-active antibiotics and a downstream amplifier of damage initiated elsewhere, a dual role that places it at the analytical center of antibiotic-induced genotoxicity.

### 4.3. Lipid Peroxidation and Protein Carbonylation

Mitochondrial ROS can attack the polyunsaturated fatty acids of inner mitochondrial and plasma membranes, generating malondialdehyde (MDA) and 4-hydroxynonenal (4-HNE). Both are reactive electrophiles capable of forming covalent DNA adducts (M1dG and ε-dA, respectively) [99,100]. 4-HNE is biologically distinctive in that it operates as a second messenger of oxidative stress, modulating NRF2 stabilization, EGFR signaling, and the apoptotic threshold, even at non-cytotoxic concentrations. The cardiolipin of the inner mitochondrial membrane is particularly susceptible to peroxidation. Oxidized cardiolipin contributes both to outer-membrane permeabilization (facilitating cytochrome c release) and to sensitization to ferroptotic cell death.

Protein carbonylation, the irreversible oxidative modification of amino acid side chains, can impair the catalytic activity of key DNA repair enzymes including OGG1 (responsible for 8-OHdG excision), APE1 (the apurinic/apyrimidinic endonuclease), and the nucleotide excision repair incision endonucleases [101]. The result is a feed-forward configuration in which oxidative stress simultaneously creates DNA lesions and impairs their resolution, a configuration that may help explain why antibiotic-induced genotoxicity often appears disproportionately severe under chronic compared to acute exposure scenarios.

### 4.4. Iron Dysregulation and Ferroptosis

Recognition of ferroptosis as an iron-dependent, lipid-peroxidation-driven form of regulated cell death has implications for antibiotic genotoxicity [22,102]. Ferroptosis is characterized by GPX4 inhibition, depletion of reduced glutathione, accumulation of phospholipid hydroperoxides, and characteristic mitochondrial morphology. Aminoglycoside-iron complexes catalyze hydroxyl radical formation at the inner mitochondrial membrane interface and can sensitize cells to ferroptotic death. Fluoroquinolone chelation of intracellular iron perturbs labile iron-pool homeostasis with similar downstream consequences [58]. The intersection of ferroptosis with DNA damage is bidirectional: oxidative DNA lesions can sensitize cells to ferroptotic death through p53-mediated SLC7A11 repression and consequent cystine import failure, while ferroptotic stress propagates DAMPs that activate inflammation and amplify genotoxicity in neighboring cells. This axis is most clearly characterized in cochlear hair cell death following aminoglycoside exposure, where ferroptosis inhibitors are protective in preclinical models. Broader relevance across mitochondria-rich tissues, cardiomyocytes, and renal proximal tubule epithelia has been proposed but is not yet supported by strong in vivo data outside the cochlear system.

### 4.5. NRF2-KEAP1 Adaptive Response

The KEAP1–NRF2–ARE axis is the dominant transcriptional response to oxidative and electrophilic stress in mammalian cells [103,104]. Under basal conditions, KEAP1 acts as a substrate adaptor for the Cullin-3-based E3 ubiquitin ligase that targets NRF2 for proteasomal degradation. Oxidative or electrophilic modification of reactive cysteine residues on KEAP1 disrupts this targeting, allowing newly synthesized NRF2 to accumulate, translocate to the nucleus, and drive transcription of antioxidant response element (ARE)-containing genes: NQO1, HO-1, GCLC, GCLM, GSR, and the xCT cystine/glutamate antiporter [103,105].

Acute antibiotic exposure typically activates NRF2 protectively. The available evidence suggests that chronic exposure can exhaust this response in two ways: depletion of the cysteine and glycine substrates required for sustained glutathione synthesis, and oxidative damage to NRF2 itself and its downstream effectors. The transition from adaptive to maladaptive NRF2 signaling defines a critical, and largely under-investigated, threshold in chronic antibiotic toxicology. The threshold’s location is likely to vary by tissue, by patient antioxidant capacity, and by concurrent pharmacological exposure [106]. Quantifying this threshold across vulnerable populations is among the more translationally consequential open questions in the field. Direct human data are sparse, and most current evidence derives from rodent and cell-culture systems.

## 5. Cytogenetic and Molecular Biomarkers

The mechanistic axes described in Section 3 and Section 4 generate detectable molecular fingerprints that are chromosomal, lesional, and transcriptomic. These fingerprints form the biomarker substrate for genotoxic surveillance in laboratory, preclinical, and human contexts. This section critically evaluates the principal assays under OECD frameworks where applicable, with emphasis on their value, limitations, and degree of convergence in antibiotic–genotoxicity research (Table 5 summarizes the principal cytogenetic and molecular biomarkers with their OECD status, endpoints detected, and key limitations). We highlight throughout the substantial inter-laboratory variability that complicates direct comparison across studies.

### 5.1. Cytokinesis-Block Micronucleus Cytome Assay (CBMN-Cyt)

**Figure 6:**
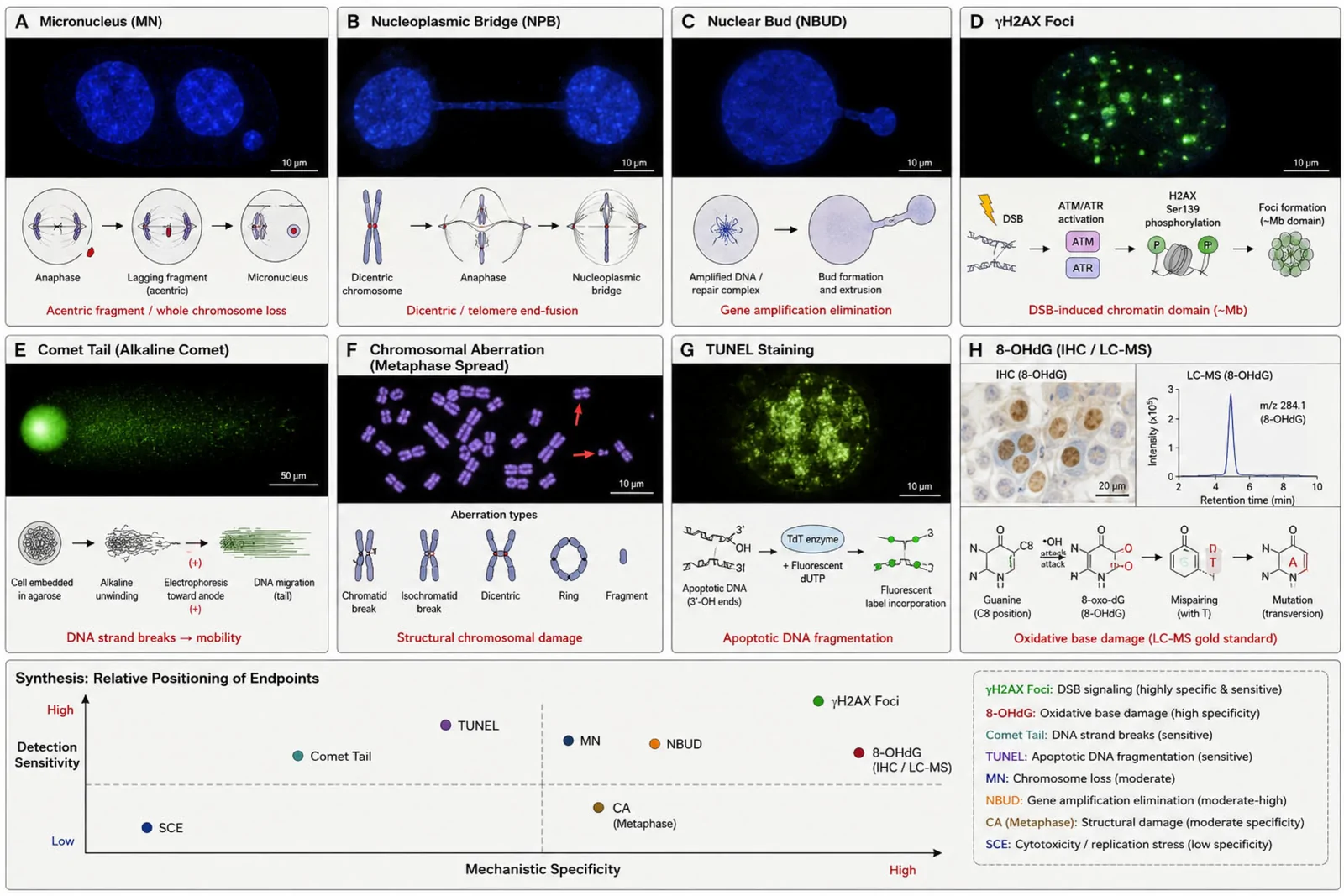
Cytogenetic and molecular genotoxicity endpoints. Panels (**A**–**H**) illustrate the principal assay endpoints used to detect antibiotic-induced DNA and chromosomal damage: micronucleus (MN), nucleoplasmic bridge (NPB), nuclear bud (NBUD), γH2AX foci, comet tail, chromosomal aberration, TUNEL staining, and 8-OHdG detection. The synthesis chart positions each endpoint by detection sensitivity and mechanistic specificity. In Panel (**F**), red arrows indicate structural chromosomal aberrations: upper arrow, chromatid break; lower arrow, acentric fragment.

The CBMN-Cyt assay is governed in regulatory contexts by OECD Test Guideline 487 (in vitro) and is widely used in human biomonitoring under HUMN and HUMN-XL project standardization [16,17,90]. It measures three cytogenetic endpoints in once-divided binucleated cells: micronuclei (arising from acentric fragments or whole chromosomes that fail to incorporate into daughter nuclei), nucleoplasmic bridges (the cytogenetic correlate of dicentric chromosome mis-segregation or telomere end-fusion), and nuclear buds (reflecting elimination of amplified DNA or unresolved repair complexes). Cytokinesis is blocked with cytochalasin-B to permit unambiguous identification of cells that have completed nuclear division. Representative photomicrographs of the three CBMN-Cyt endpoints, alongside comet tails and γH2AX foci that anchor the assays discussed in Section 5.2 and Section 5.5, are presented in Figure 6.

Strengths of the assay include simultaneous capture of clastogenic and aneugenic events within a single framework, scalability through automated scoring platforms, and the availability of extensive historical control data through the HUMN project. The limitations are equally important. CBMN-Cyt is relatively insensitive to lesions that do not progress to mitotic mis-segregation. It is susceptible to cytotoxicity confounding, since high cytotoxicity can artefactually elevate or suppress MN frequency. Cytochalasin-B itself exerts a small but non-zero effect on chromosome behavior. Inter-laboratory variability in scoring criteria has been documented even within the HUMN consortium. CBMN-Cyt has produced reproducibly positive signals for ciprofloxacin and metronidazole at clinically relevant concentrations in human lymphocytes [39,40]; results for β-lactams, macrolides, and tetracyclines are more variable, partly reflecting genuine class differences and partly methodological heterogeneity.

### 5.2. Comet Assay (Single-Cell Gel Electrophoresis)

The alkaline and neutral variants of the single-cell gel electrophoresis (comet) assay detect DNA strand breaks at the level of individual cells through the electrophoretic mobility of fragmented DNA. The in vivo formulation is governed by OECD Test Guideline 489 [18,107]. The alkaline variant detects both single- and double-strand breaks (and, with Fpg or OGG1 digestion, oxidatively modified bases). The neutral variant is selective for DSBs. The assay is exceptionally sensitive, capable of detecting fewer than 100 strand breaks per cell, requires only thousands of cells per condition, and is amenable to high-throughput formats.

Limitations are well documented. Inter-laboratory variability in scoring (% tail DNA, tail moment, and Olive moment) complicates cross-study comparison. Electrophoresis duration, voltage, alkali unwinding time, and image-analysis software thresholds all influence outcomes. The Minimum Information for Reporting on the Comet Assay (MIRCA) standards address part of this problem by mandating disclosure of methodological detail [35], but adherence remains uneven. Fluoroquinolone- and metronidazole-induced comet signals are among the most reproducible in the antibiotic–genotoxicity literature, though signal magnitude varies meaningfully across laboratories even at comparable concentrations. Recent in vivo applications have extended the assay to additional clinical contexts: intravesical tigecycline administration in a rat cystitis model produced dose-independent comet-detectable DNA damage in bladder tissue across both therapeutic and sub-therapeutic dose groups, illustrating the assay’s utility under realistic therapeutic regimens [76].

### 5.3. Chromosomal Aberration Assay

OECD Test Guidelines 473 (in vitro) and 475 (in vivo bone marrow) prescribe the structural chromosomal aberration assay, in which metaphase-arrested cells are scored for chromatid- and chromosome-type aberrations: breaks, gaps, fragments, dicentrics, rings, and exchanges [108]. The assay provides direct visualization of clastogenic events and remains a regulatory benchmark. It is, however, labor-intensive, requires expert cytogenetic scoring, and is less sensitive than γH2AX-based methods for low-dose exposures. The pooled cohort analysis of Bonassi and colleagues demonstrated that baseline chromosomal aberration frequency in peripheral lymphocytes is independently associated with subsequent cancer risk in healthy populations, validating chromosomal aberration as a biologically meaningful endpoint with long-term predictive value at the population level [109].

### 5.4. Sister Chromatid Exchange

Sister chromatid exchange (SCE) detects homologous recombination events between sister chromatids, visualized after bromodeoxyuridine incorporation and differential staining. Once a workhorse of genetic toxicology, SCE has fallen into relative disuse because its mechanistic interpretation is ambiguous: elevated SCE indicates recombinational activity but does not unambiguously indicate genotoxic damage [110]. Nevertheless, in antibiotic biomonitoring studies, SCE retains value as corroborative evidence, particularly when historical comparability with the extensive cytogenetic surveys of the 1970s and 1980s is informative.

### 5.5. γH2AX Assay

Phosphorylation of histone H2AX on Ser139 at sites of DSBs is detected by immunofluorescence microscopy (foci counting) or by flow cytometry (mean fluorescence intensity), with effective single-DSB sensitivity [14,85,111]. Over the past fifteen years, the γH2AX assay has become the most widely used molecular biomarker of genotoxic DSB induction in vitro and is applied in clinical biomonitoring. The limitations include the persistence of γH2AX in apoptotic cells, which requires co-staining with cleaved caspase-3 or annexin V to discriminate primary damage from terminal fragmentation, and substantial inter-laboratory variability in foci-detection thresholds [112]. The blood-drop method described by Heylmann and Kaina permits γH2AX quantification from sub-milliliter blood samples, partially addressing the practical constraints of pediatric biomonitoring [113]. Although γH2AX currently lacks a formal OECD Test Guideline, its proposed use as a regulatory pre-screen has gained momentum within the new approach methodologies (NAMs) framework.

### 5.6. TUNEL Assay

Terminal-deoxynucleotidyl-transferase dUTP nick-end labeling (TUNEL) detects 3′-OH ends generated during apoptotic DNA fragmentation [114]. It is mechanistically distinct from primary genotoxicity assays and is best interpreted as a marker of the downstream apoptotic consequence of genotoxic injury rather than of the initiating genotoxic event. In antibiotic studies, TUNEL is most informative when read alongside γH2AX, allowing discrimination of pre-apoptotic genome injury (γH2AX^+^/TUNEL^−^) from terminal fragmentation (γH2AX^+^/TUNEL^+^).

### 5.7. Oxidative and Mitochondrial Biomarkers

Multiple oxidative and mitochondrial biomarkers complement the cytogenetic endpoints described above (Table 5). 8-Hydroxy-2′-deoxyguanosine (8-OHdG) is the most widely used biomarker of oxidative DNA damage, quantifiable in urine, leukocyte DNA, or tissue homogenates [36,115]. Ultra-high-performance liquid chromatography-tandem mass spectrometry (UHPLC-MS/MS) provides the gold-standard quantification. Commercial ELISA kits offer high throughput but suffer from cross-reactivity issues that can produce systematic overestimation. Malondialdehyde and 4-HNE are markers used to quantify lipid peroxidation through TBARS, HPLC, and LC-MS/MS approaches; TBARS results in particular are subject to substantial confounding and should be interpreted cautiously. The glutathione redox ratio (GSH/GSSG) reports the cellular thiol redox state but is highly sensitive to sample handling. Mitochondrial endpoints include the mtDNA copy number (qPCR ratio against nuclear DNA), the common 4977-bp mtDNA deletion, and ΔΨm assessment by JC-1 or TMRM. No single biomarker captures the integrated genotoxic–oxidative state of an exposed cell or tissue, and a multi-marker panel approach is now standard practice in mechanistic studies.

### 5.8. Convergent Positivity as Evidentiary Standard

No single assay constitutes a sufficient genotoxic endpoint for causal inference in antibiotic studies. Each assay captures a particular biological dimension: cytogenetic outcome (CBMN), strand-break burden (comet), structural damage (CA), recombinational activity (SCE), DSB initiation (γH2AX), apoptotic execution (TUNEL), or oxidative–mitochondrial state. Each is subject to characteristic confounders. The most defensible evidentiary standard for causal inference is cross-assay positivity across mechanistically distinct assays, ideally including at least one in vitro genotoxicity endpoint (comet or MN), one molecular DSB marker (γH2AX), and one oxidative or mitochondrial endpoint (8-OHdG or ΔΨm). Where antibiotic studies report positive signals in only one assay, mechanistic interpretation should be correspondingly cautious, and replication across model systems should be a precondition for translational extrapolation. A substantial fraction of the antibiotic–genotoxicity literature does not meet this convergence standard, which limits the strength of broader generalizations.

## 6. Epigenetic and Transcriptomic Alterations

Beyond direct lesion formation and DDR engagement, antibiotic exposure perturbs the regulatory landscape of the genome (DNA methylation, histone modifications, and non-coding RNA networks). These changes may, in principle, translate acute exposure into long-term programming risk. The strength of evidence for this translational claim is uneven, and we discuss it accordingly.

### 6.1. DNA Methylation

Antibiotic exposure has been reported to modulate DNA methylation at both global and locus-specific levels in mammalian cells. Global hypomethylation, particularly at repetitive elements (LINE-1 and Alu), is associated with chromosomal instability and has been observed following chronic exposure to several antibiotic classes in vitro [116]. Locus-specific hypermethylation can silence tumor suppressor genes (CDKN2A and RASSF1A) and DNA repair genes (MLH1 and MGMT), generating a permissive epigenetic state for cumulative mutational burden [117]. Proposed mechanistic bases include oxidative damage to 5-methylcytosine (generating 5-hydroxymethylcytosine and downstream demethylation intermediates), perturbation of TET-mediated demethylation by mitochondrial dysfunction (which depletes α-ketoglutarate, an obligatory TET cofactor), and direct effects of oxidative stress on DNMT activity and substrate availability [118,119].

Perinatal antibiotic exposure has been associated with differential DNA methylation at developmentally regulated imprinted loci (Igf2, H19, Meg3, Peg3, and Plagl1) in cord-blood DNA, suggesting that the developing methylome is particularly vulnerable [70,71]. These associations are observational, derived from cohorts with modest sample sizes, and have not always been replicated. Confounding by indication is a serious concern: the underlying maternal or neonatal infection may itself drive methylation differences independent of antibiotic exposure. Until replication in larger, infection-controlled cohorts is achieved, these findings should be regarded as hypothesis-generating rather than causal.

### 6.2. Histone Modifications

Beyond γH2AX, antibiotic exposure has been reported to alter the abundance and distribution of additional histone marks that influence DNA repair accessibility and transcriptional programs. H3K9me3 (heterochromatin) and H4K20me3 redistribute during oxidative stress, modulating chromatin compaction at damage sites. H3K4me3 (active promoters) and H3K27ac (enhancers) shift in patterns that reflect transcriptional reprogramming of stress-response and inflammatory gene networks [120]. The histone-modifying enzymes that drive these changes (histone deacetylases, lysine methyltransferases, and lysine demethylases) depend on mitochondrial cofactors (NAD^+^ for sirtuins, α-ketoglutarate for KDMs), which tightly couples chromatin state to bioenergetic status and provides a mechanistic link between mitochondrial dysfunction and epigenetic perturbation [119], though most direct evidence for antibiotic-specific histone effects comes from in vitro work in immortalized cell lines.

### 6.3. Non-Coding RNAs

Non-coding RNAs respond to antibiotic-induced oxidative and DDR signaling and may serve as both effectors and biomarkers of genotoxic stress. miR-21 is induced by oxidative stress and suppresses pro-apoptotic targets, including PDCD4. miR-34a is a direct p53 transcriptional target and amplifies apoptotic and senescence programs. miR-155 mediates inflammatory cytokine signaling and has been reported to be elevated in tissues experiencing chronic genotoxic stress [121,122]. Long non-coding RNAs (NORAD and GUARDIN) regulate genomic stability and telomere integrity, and their dysregulation has been documented in oxidatively stressed cells. These ncRNAs are stably detectable in plasma, exosomes, and other extracellular compartments, offering accessible biomarker potential for human biomonitoring, particularly relevant in pediatric contexts where invasive sampling is constrained. The application of ncRNA biomarkers to antibiotic genotoxicity specifically remains preliminary, and standardization of preanalytical handling is a known concern.

### 6.4. Transcriptomic Signatures

Genome-wide RNA-seq of antibiotic-exposed cells has consistently identified enrichment of DDR signaling (p53, ATM/ATR, and BRCA), oxidative stress response (NRF2 targets), the mitochondrial unfolded protein response (UPRmt: HSP60, CLPP, and LONP1), and inflammatory pathways (NF-κB and type I IFN). These signatures provide mechanistic granularity that single-endpoint assays cannot match. The TGx-DDI transcriptomic biomarker, developed by Yauk and colleagues at Health Canada and now in regulatory consideration, classifies DNA damage-inducing chemicals through a 64-gene panel with substantial accuracy across diverse chemical classes [123]. Its application to antibiotic genotoxicity is an actively developing area but has not yet been formally validated for the six prioritized classes.

### 6.5. Developmental and Transgenerational Considerations

The plasticity of the early-life epigenome, combined with the high frequency of antibiotic exposure during infancy and early childhood, raises a biologically reasonable but empirically underpowered hypothesis: long-term epigenetic programming with later-life health consequences. The proposed mechanism is straightforward in outline. Developmental windows of high epigenome plasticity, from in utero through early postnatal life, intersect with the period of highest cumulative antibiotic exposure. Epigenetic marks established during these windows may persist through subsequent cell divisions and influence later-life metabolic, immune, and oncologic risk. Cohort studies have begun to detect signals at imprinted and metabolically relevant loci [70,71]. Establishing causality between early-life antibiotic exposure and later-life disease through epigenetic mediation remains, however, a major translational challenge. Confounding by indication, by underlying microbial exposure, and by family-level socioeconomic factors all complicate inference. Current evidence supports continued investigation but does not yet support strong causal claims.

## 7. Pediatric, Adult, and Other Vulnerable Populations

The mechanisms developed in Section 3, Section 4, Section 5 and Section 6 do not operate uniformly across humans. Susceptibility is set by developmental pharmacokinetics in the young, by declining repair capacity and renal clearance in the elderly, by pharmacogenomic variation in metabolism and DNA repair across all ages, and—for several populations—by the simple absence of dedicated clinical pharmacovigilance data. Section 7 takes the major susceptibility strata one at a time. The candid summary is that mechanistic argumentation for elevated risk in these populations rests on strong biological premises but on essentially no dedicated human biomonitoring data: fewer than 5% of cytogenetic biomonitoring studies include pediatric subjects, and pregnancy-specific studies are essentially absent. Strong policy claims for these groups are, therefore, biologically motivated rather than empirically established, and we mark them accordingly throughout.

### 7.1. Developmental Pharmacokinetics and Immature DNA Repair

Neonates and infants exhibit markedly different drug-handling characteristics from adults. Hepatic phase I enzyme activity is immature: CYP3A4 expression reaches adult levels only over the first 1–2 years of life, and CYP1A2 matures over a similar period. CYP3A7, a fetal-predominant enzyme, persists into early infancy [32,124]. Phase II conjugation (glucuronidation and sulfation) is similarly developmentally regulated. Renal clearance is approximately 30–40% of adult capacity at birth and matures to adult function over the first year [125]. Hepatic and renal immaturity together result in higher peak tissue concentrations and longer elimination half-lives for many antibiotics in pediatric patients, narrowing the genotoxic margin.

Glutathione reserves are lower in neonates and phase II conjugating-enzyme activity is reduced. DNA repair kinetics differ qualitatively rather than only quantitatively. Pediatric cells in proliferating tissues use non-homologous end joining (NHEJ) as the dominant DSB repair pathway; homologous recombination (HR), the higher-fidelity option, requires S-phase availability of a sister chromatid template that is more reliably present in slowly cycling adult tissues [126]. The mechanistic prediction is that the same DSB load translates into a higher mutation–fixation probability in pediatric tissue. The clinical context heightens the concern: fluoroquinolones are used off-label in pediatric oncology, cystic fibrosis, and serious gram-negative infections, populations in which the host is already under genotoxic load from underlying disease and concurrent therapy. Direct cytogenetic biomonitoring in pediatric antibiotic-exposed cohorts is essentially absent. No dedicated cohort has measured γH2AX, MN, or 8-OHdG kinetics in pediatric patients during and after antibiotic treatment with infection-only controls. This is the clearest example in the field of a strongly biologically motivated risk claim that the human data have not yet been collected to support or refute.

### 7.2. Human Biomonitoring Evidence and Its Limits

Human biomonitoring of antibiotic genotoxicity is dominated by small single-center investigations using peripheral blood lymphocyte CBMN-Cyt or comet endpoints, with sample sizes rarely exceeding 50–100 exposed individuals per study and infection-only control arms uncommon. The metronidazole literature is the most developed: studies in adult cohorts under therapeutic dosing have reported modest elevations in MN frequency (typically 1.5- to 3-fold above baseline) and comet tail moment during therapy, with normalization within 4–6 weeks of treatment cessation [9,39,40]. The fluoroquinolone biomonitoring literature is genuinely conflicting—positive cytogenetic signals have been reported in some adult cohorts under short-course ciprofloxacin or levofloxacin and equivocal results in others, with effect sizes that do not consistently scale with cumulative dose. β-Lactam biomonitoring is consistently negative for direct cytogenetic endpoints, which is mechanistically expected given that this class operates primarily through microbiome-mediated indirect routes that lymphocyte assays do not capture. Occupational data from pharmaceutical-industry workers handling antibiotic active pharmaceutical ingredients show small but detectable cytogenetic elevations across several classes; translational extrapolation to therapeutic exposure is limited because occupational exposure differs in route (inhalation/dermal), pattern (chronic low-level), and duration (years). Table 6 summarizes the available biomonitoring evidence and the critical gaps across principal vulnerable populations. The structural problem in this literature is not absent evidence but consistent under-powering and lack of infection-only controls; meta-analytic synthesis cannot resolve heterogeneity that arises from inadequate primary study design.

A persistent methodological gap is the absence of large, infection-controlled, multi-center pediatric cohorts. Children represent the demographic with the highest lifetime cumulative antibiotic exposure but are represented in fewer than 5% of antibiotic cytogenetic biomonitoring studies in the published literature. Pregnancy biomonitoring is essentially absent. Until this gap is addressed through dedicated cohort design that uses minimal-volume γH2AX or comet methodologies suitable for pediatric venipuncture or cord-blood biobanking, strong claims about pediatric or pregnancy-specific risk should be regarded as biologically motivated rather than empirically established.

### 7.3. Polypharmacy and Chronic Exposure in Adults

Adult patients with chronic conditions routinely receive antibiotics concurrently with agents that share or amplify the genotoxic axes developed in this review. NSAIDs elevate oxidative stress and impair gastric and intestinal mucosa repair; statins perturb mitochondrial function through coenzyme Q depletion; proton-pump inhibitors reshape the microbiome with downstream effects on bile-acid genotoxicity; and SSRIs influence platelet and mitochondrial function. The combinatorial genotoxic burden of these co-exposures is essentially uncharacterized. To our knowledge, no factorial in vitro design has measured cytogenetic endpoints across an antibiotic and a chronic-disease comedication, and no pharmacoepidemiologic cohort has stratified antibiotic-associated cytogenetic outcomes by concurrent statin, NSAID, or PPI use. The combinatorial scenarios most likely to matter clinically—aminoglycoside plus loop diuretic in critical care, fluoroquinolone plus NSAID in renal-compromised patients, and macrolide plus statin in cardiovascular disease—are precisely the ones the current biomonitoring infrastructure is least equipped to detect. This is one of the more tractable methodological gaps identified in this review: factorial in vitro designs are technologically feasible with current cytogenetic platforms, and pharmacoepidemiologic databases support stratified analysis at the population level. The barrier is institutional commitment, not technical capability.

Geriatric patients additionally accumulate genotoxic burden from declining DNA repair fidelity (age-related reduction in OGG1, APE1, and BER capacity), impaired antioxidant defense, and renal clearance reductions that prolong tissue antibiotic exposure [127]. The intersection of pharmacokinetic and biological aging is most consequential for aminoglycosides, whose renal accumulation in elderly patients elevates both nephrotoxicity and ototoxicity risk. Dose adjustment based on creatinine clearance partially mitigates this concern, though therapeutic drug monitoring is not uniformly available in all clinical settings.

### 7.4. Pregnancy and Oncology Populations

Pregnancy intersects antibiotic exposure with embryonic and fetal cells whose proliferation rates and reliance on faithful chromosome segregation are at their developmental peak. Transplacental exposure to fluoroquinolones and metronidazole has historically been restricted on a precautionary basis, although large pharmacoepidemiologic studies have not consistently demonstrated a teratogenic signal at standard therapeutic doses [128]. Mechanistically, the case for caution remains coherent: fetal hematopoietic and neural progenitors are precisely the cell types most sensitive to Topo II–mediated genotoxicity. Empirically, the absence of a teratogenic signal in available cohorts is reassuring but does not address potential subtle developmental effects that would not be detected by standard pharmacoepidemiologic endpoints.

In oncology, antibiotic prophylaxis is routine in neutropenic patients and surgical cancer care. Additive or synergistic genotoxicity with concurrent cytotoxic chemotherapy or radiation has been mechanistically inferred but rarely formally measured. The dominant chemotherapeutic damage signal typically obscures antibiotic-specific contributions in clinical biomonitoring, and dedicated study designs would be required to disentangle them. Other vulnerable populations include patients with cystic fibrosis on lifelong aminoglycoside cycling, transplant recipients on chronic immunosuppression with concurrent prophylaxis, and HIV patients with chronic comorbidity-driven antibiotic exposure. Each of these populations represents a distinct exposure context in which the cumulative genotoxic burden warrants more systematic characterization than currently exists.

### 7.5. Pharmacogenomic Modulators of Individual Susceptibility

Inter-individual variation in antibiotic genotoxicity is partly mediated by polymorphisms in drug metabolism and DNA repair. The metabolic side has the better-developed evidence base. CYP1A2 and CYP2C19 polymorphisms alter clearance of several antibiotic substrates, CYP3A4/5 polymorphisms influence macrolide and fluoroquinolone disposition, and m.1555A>G in mitochondrial 12S rRNA confers near-complete penetrance for aminoglycoside ototoxicity—the single best-characterized antibiotic–pharmacogenomic interaction in clinical use, with pre-treatment screening recommendations already implemented in several health systems. The repair-pathway side is weaker. GSTM1 and GSTT1 null genotypes reduce glutathione-S-transferase capacity and have been associated with an elevated baseline and induced cytogenetic damage across multiple chemical classes [129], but antibiotic-specific stratified data are sparse. OGG1 Ser326Cys and XRCC1 Arg399Gln modulate base excision repair of oxidative lesions [130]; their effect on antibiotic-specific endpoints has not been formally characterized. MTHFR C677T influences folate-mediated nucleotide synthesis and methylation, but its antibiotic–genotoxicity relevance is unstudied. Outside the m.1555A>G case, pharmacogenomically stratified antibiotic prescribing is not implemented anywhere on genotoxicity grounds. The evidence base would require coordinated multi-center cohorts before such implementation could be defended on cost-effectiveness or clinical-utility grounds. This is not an objection in principle; it is a statement about what evidence is currently in hand.

## 8. Clinical and Public Health Implications

Translating the mechanistic and biomarker evidence into clinical practice and policy requires calibration along three dimensions: antibiotic selection in high-stakes populations, the framing of antimicrobial stewardship, and regulatory genotoxicity assessment paradigms. Section 8 takes these in turn, with the explicit acknowledgement that for most antibiotic classes the mechanistic case currently outpaces clinical-endpoint evidence—and that this asymmetry is itself a policy condition that warrants explicit recognition rather than rhetorical hedging.

### 8.1. Carcinogenicity Signals and Regulatory Classifications

Metronidazole’s IARC Group 2B classification was established in 1987 on the basis of robust rodent carcinogenicity and mechanistic plausibility [53]. The classification has not been revisited. Four decades of pharmacoepidemiology have not identified consistent excess cancer incidence in exposed populations [54], although most available studies are underpowered for long-latency outcomes and confounded by indication—patients prescribed metronidazole differ systematically from the general population in ways that could affect cancer risk independent of drug exposure. The principled options for IARC are three: (i) downgrade to Group 3 (not classifiable) on the basis of negative human data, (ii) maintain Group 2B with explicit acknowledgement that the human signal has not emerged in four decades of monitoring, or (iii) commission a re-evaluation that integrates contemporary mechanistic and pharmacoepidemiologic evidence. The status quo of an unrevisited 1987 classification is the least defensible of the three.

Fluoroquinolone associations with aortic aneurysm and dissection [47], tendinopathy [48], peripheral neuropathy, and the broader fluoroquinolone-associated disability syndrome [49] are documented at the population level and have prompted FDA black-box warnings and the recommendation that fluoroquinolones be restricted to infections without effective alternatives. The carcinogenic implications of chronic or repeated exposure are mechanistically non-trivial given the Topo II liability shared with established Topo II–poison carcinogens, such as etoposide. Human epidemiology has not identified a comparably strong excess malignancy signal: available cohort and case-control studies have produced sparse and inconsistent associations that do not currently support Group 2B classification on epidemiologic grounds. The mechanistic case is sufficient to warrant systematic IARC re-examination but not sufficient to predict the outcome. A defensible regulatory position is to maintain current restrictions on fluoroquinolone use to indications without alternatives while commissioning long-term cohort follow-up of large fluoroquinolone-exposed populations with cancer endpoints—work that is technologically feasible with existing pharmacoepidemiologic databases.

### 8.2. Antimicrobial Stewardship Reframed

Antimicrobial stewardship has historically been framed around resistance prevention at the population level. The mechanistic case developed in this review supports an expanded framing in which the genotoxic burden on the individual host—particularly the pediatric host—is also weighed. This expansion does not alter prescribing for clearly indicated infections, where the benefit–risk calculation is dominated by the consequences of untreated infection. It does counsel against marginal indications and prophylactic regimens in pediatric, neonatal, and pregnancy contexts; it supports class-selection algorithms that account for the comparative genotoxic profiles of equivalently effective agents. In practical terms: a child with otitis media for whom amoxicillin and a fluoroquinolone are both effective should receive amoxicillin not only on resistance-stewardship grounds but on genotoxic-stewardship grounds. The choice is informed by the genotoxic-profile differential developed in Section 2, even though that differential has not been directly measured in pediatric cohorts.

The WHO AWaRe classification (Access, Watch, and Reserve) [131] provides a partial framework for this expansion but is currently organized around resistance considerations alone. Aligning AWaRe priorities with genotoxic considerations—for example, elevating concern for fluoroquinolone use in pediatric patients beyond resistance grounds—could be examined as a potential future component of stewardship frameworks, contingent on clinical-endpoint validation. Whether genotoxic considerations should be formally incorporated into Watch and Reserve criteria is a question for future WHO Expert Committee review, and such incorporation would require stronger clinical evidence than is currently available. The argument should be made with explicit acknowledgement of evidence limits: mechanistic considerations should inform stewardship policy but should not exclusively drive it until clinical-endpoint validation has matured for the classes in question.

### 8.3. The Environmental and One Health Dimension

Antibiotic residues in surface water, drinking water, soil, and the food chain expose human populations to chronic low-dose antibiotic exposures whose cumulative genotoxic implications are difficult to quantify but biologically reasonable [132,133]. The veterinary use of antibiotics, particularly in livestock production, contributes substantially to the environmental antibiotic burden and to indirect human exposure through the food chain. Environmental monitoring of antibiotic residues is now well established in the European Union and increasingly elsewhere, but the integration of environmental antibiotic exposure data with human biomonitoring of genotoxic endpoints remains in its infancy. A One Health framework that integrates human, veterinary, and environmental antibiotic exposure with genotoxic biomonitoring represents an emerging priority that aligns the antibiotic–genotoxicity field with broader environmental health science.

### 8.4. Risk–Benefit Calibration

Nothing in this review argues against antibiotic use where clinically indicated. Bacterial infection remains a major global cause of morbidity and mortality, and antibiotic therapy has saved enormous numbers of lives; this point bears explicit reaffirmation precisely because mechanistic discussions of antibiotic toxicity can otherwise be read as anti-antibiotic. The argument is for proportionate use along four dimensions. First, the vigilant monitoring of vulnerable populations through dedicated cytogenetic biomonitoring infrastructure that does not currently exist at scale. Second, the expansion of clinical pharmacovigilance to include genotoxic endpoints where the evidence supports inclusion—the m.1555A>G–aminoglycoside interaction is the closest to clinical implementation, fluoroquinolone tendon and aortic surveillance is partially implemented, and carcinogenicity surveillance is essentially absent. Third, the integration of mechanistic insight into individualized prescribing, where genotype, age, or pregnancy status alters the benefit–risk balance materially. Fourth, the alignment of stewardship priorities with host genome protection rather than resistance prevention alone. In the highest-stakes contexts—pediatric oncology, neonatal intensive care, pregnancy, and chronic prophylaxis—the case for genotoxically informed antibiotic selection is mechanistically supported and operationally feasible with existing tools; the regulatory and clinical infrastructure to systematize such selection is buildable rather than aspirational. The absence of strong direct human evidence for many of the proposed risks should temper the strength of policy recommendations, but it should not be permitted to defer the methodological investments that would generate the missing evidence.

## 9. Methodological Limitations and Research Gaps

Critical interpretation of the antibiotic–genotoxicity literature requires explicit attention to several methodological tensions. We catalogue these in this section rather than dispersing them across the manuscript, although the relevant caveats have been signaled throughout. Five principal limitations constrain interpretation of the available evidence; Table 7 expands these into ten specific methodological gaps with their tractable approaches and realistic time horizons.

### 9.1. The In Vitro–In Vivo–Human Extrapolation Problem

A persistent tension in antibiotic–genotoxicity research is the gap between concentrations producing measurable in vitro effects and clinically achievable plasma concentrations. In vitro studies of ciprofloxacin, metronidazole, and azithromycin frequently report positive cytogenetic signals at concentrations one to two orders of magnitude above peak therapeutic plasma levels [43,64]. This gap is not necessarily disqualifying. Tissue accumulation in the renal cortex, cochlear hair cells, hepatic mitochondria, and macrophage compartments can produce intracellular concentrations approaching or exceeding the active in vitro range, particularly for drugs with high tissue-to-plasma ratios (azithromycin) or active transport-mediated accumulation (aminoglycosides through megalin/cubilin). Nevertheless, the field has not adopted a uniform standard for relating in vitro test concentrations to clinically relevant tissue exposure, and physiologically-based pharmacokinetic (PBPK) modelling, which could bridge this gap, remains underutilized in genotoxicity study design.

Equally important is the heavy reliance on immortalized cell lines (HeLa, V79, CHO, TK6, and HepG2). These lines harbor genomic and metabolic alterations that may not faithfully recapitulate primary-cell repair capacity, mitochondrial bioenergetics, or stress-response thresholds. Genotoxic signals observed in TK6 cells, for example, can exceed or differ qualitatively from those obtained in primary human lymphocytes under matched exposure conditions. Increased use of primary cells, iPSC-derived models, and microphysiological systems (Section 10.2) would address this limitation but requires substantially more investment than acute cell-line screening.

Exposure duration is another concern. Most published in vitro genotoxicity studies use exposures of 3–24 h. Real-world antibiotic exposure spans days for acute courses to months or years for chronic dermatologic, prophylactic, or suppressive regimens. The mechanistic processes likely differ qualitatively across these timescales: acute exposure favors direct lesion induction and acute DDR engagement, whereas chronic exposure may favor cumulative mitochondrial decline, epigenetic drift, and senescence accumulation. Standardized chronic-exposure paradigms for in vitro genotoxicity are not well developed for antibiotics.

### 9.2. Infection as Confounder in Human Biomonitoring

The single most consequential confounder in human biomonitoring of antibiotic genotoxicity is the infection itself. Bacterial and viral infections independently elevate systemic oxidative stress, induce inflammatory cytokine cascades, and produce measurable elevations in 8-OHdG, micronucleus frequency, and γH2AX foci [134,135]. Studies that compare antibiotic-treated patients to healthy controls, a common design, necessarily conflate drug effect with infection effect. The methodologically rigorous alternative is paired sampling at pre-treatment, on-treatment, and post-treatment time points, ideally with an infection-only control arm. Such designs are ethically feasible only in mild, self-limiting conditions, and they remain rare in the existing literature. The absence of this design is a principal reason why mechanistically robust in vitro signals do not always translate into demonstrable human cytogenetic damage.

Species differences further complicate translation. Rodent models, the dominant in vivo system in the antibiotic–genotoxicity literature, differ from humans in mitochondrial copy number per cell, antioxidant enzyme stoichiometry, DNA repair allocation between HR and NHEJ, and pharmacokinetic profile. Pediatric extrapolation is particularly difficult: juvenile rodent toxicology does not straightforwardly map to human pediatric exposure. Interindividual variability within human populations is driven by pharmacogenomics, baseline antioxidant capacity, comorbidity, microbiome composition, and concurrent medications, all of which further widens the range of plausible responses to a given antibiotic exposure.

### 9.3. Assay Standardization Across Laboratories

Despite OECD harmonization frameworks for the cytokinesis-block micronucleus cytome assay (TG 487) and the comet assay (TG 489), inter-laboratory variability in scoring criteria, electrophoresis conditions, and foci-detection thresholds remains substantial. The HUMN and HUMN-XL international validation projects have made significant progress for the micronucleus assay, generating extensive historical control databases and identifying key sources of inter-laboratory variation [136]. The MIRCA reporting standards for the comet assay address the multiplicity of permissible readouts by mandating disclosure of methodological detail [35], though adherence is uneven and pre-MIRCA studies are difficult to compare directly. The γH2AX detection threshold standardization remains less developed. Inter-platform comparison is an active methodological need. Ring-trial validation of these assays specifically for antibiotic genotoxicity testing would close a substantial portion of this gap.

### 9.4. Underrepresentation of Pediatric, Pregnancy, and Other Vulnerable Cohorts

The pediatric biomonitoring deficit, signaled throughout this review, deserves restatement here. Children represent the demographic with the highest lifetime cumulative antibiotic exposure, the most plastic epigenome, and the most active proliferating tissue compartments, yet are represented in fewer than 5% of antibiotic cytogenetic biomonitoring studies. Pregnancy biomonitoring is essentially absent. Ethical and regulatory constraints, particularly the requirement that biomonitoring not impose more than minimal risk in pediatric populations, partly explain this deficit. Technological solutions exist: the γH2AX blood-drop method permits damage quantification from sub-milliliter blood samples [113], and opportunistic biobanking of routinely collected pediatric blood and cord blood would generate the necessary sample base without imposing additional procedures on patients. Multi-center collaboration through pediatric oncology and neonatology networks offers a realistic pathway to closing this gap within five to ten years. Until this gap is addressed, pediatric-specific risk claims should be regarded as biologically motivated rather than empirically established.

### 9.5. Mixed-Drug and Microbiome-Mediated Exposure

Real-world antibiotic exposures occur within complex pharmacological contexts: combination antibiotic therapy, concurrent non-antibiotic medications, and concurrent perturbation of the gut microbiome. The combinatorial genotoxicity of these scenarios is almost entirely uninvestigated, yet several mechanistically grounded predictions follow directly. Aminoglycoside-plus-NSAID combinations might produce additive renal oxidative stress. Fluoroquinolone-plus-glucocorticoid combinations might elevate tendinopathy risk through additive Topo IIβ effects. β-Lactam-plus-PPI combinations might amplify microbiome-mediated bile-acid genotoxicity in colonocytes. Each of these predictions is empirically testable through factorial study designs, gnotobiotic models, and pharmacoepidemiologic database analyses. The current absence of such investigations reflects, in part, the historical organization of the field around single-drug studies rather than around realistic exposure contexts.

## 10. Emerging Technologies and Future Perspectives

The methodological limitations catalogued in Section 9 are not permanent. Five emerging technological domains have the potential to address the most critical gaps within a five-to-ten-year horizon (Table 8 catalogues these technologies with their current readiness and principal limitations). We discuss each below, while remaining alert to the substantial gap between technological availability and clinical or regulatory implementation. Enthusiasm about emerging methodologies should be balanced against the time scale on which they have historically translated to standard practice.

### 10.1. Multi-Omics Integration

Integration of transcriptomic, epigenomic, metabolomic, and proteomic data on antibiotic-exposed primary cells and tissues offers a direct route to mechanistically resolved class-specific signatures. The TGx-DDI transcriptomic biomarker, developed by Yauk and colleagues at Health Canada, provides a 64-gene panel that classifies DNA damage-inducing chemicals with substantial accuracy across diverse chemical classes [123,137]. Its application to antibiotic genotoxicity is a near-term opportunity: the existing panel could in principle be applied to in vitro antibiotic exposure datasets, generating class-specific molecular signatures that refine hazard ranking and identify mechanistic similarities and divergences across the six prioritized classes. Joint multi-omics analyses extending beyond transcriptomics to incorporate epigenome, metabolome, and lipidome would further refine these signatures, particularly for the chronic-exposure scenarios that dominate real-world antibiotic use but are poorly represented in acute high-dose toxicology. The principal practical constraints are cost, the requirement for harmonized cell systems, and the analytical complexity of integrating heterogeneous data streams.

### 10.2. Microphysiological Systems and Organoids

Microphysiological systems (organ-on-chip platforms with continuous flow, multi-cell-type co-culture, and physiologic shear stress) recapitulate tissue-specific pharmacokinetics more faithfully than conventional 2D cell culture [138]. Kidney-on-chip platforms reproduce proximal tubule reabsorption and aminoglycoside accumulation. Liver-on-chip platforms incorporate phase I and II metabolism and bile-canalicular function. Gut-on-chip platforms permit co-culture with microbiome species and modeling of β-lactam-induced dysbiosis [139]. iPSC-derived organoids of kidney, cochlear, hepatic, cardiac, and intestinal tissue allow patient-specific modeling of antibiotic susceptibility, with particular value for pediatric and pregnancy contexts that resist direct human study. The regulatory acceptance of MPS-derived data for hazard identification has progressed within the NAMs framework. The FDA Modernization Act 2.0 (2022) explicitly permits MPS-derived data in support of regulatory submissions [140]. The technology is no longer purely experimental, though widespread integration into routine genotoxicity testing remains years away.

Realistic expectations are important. Organoid and MPS platforms are technically demanding, expensive, and have not yet been standardized for cross-laboratory comparability in genotoxicity contexts. Their adoption will likely proceed in stages: first for mechanistic discovery, later for regulatory hazard identification, and only eventually for routine prescribing-decision support.

### 10.3. Artificial Intelligence and Machine Learning

AI and machine learning approaches span several genotoxicity-relevant tasks: QSAR for chemical-space exploration of hazard liability, deep-learning classifiers trained on DDR transcriptomic signatures, automated γH2AX foci scoring at high-content imaging throughput, and large-language-model-curated literature synthesis [141,142]. For antibiotic genotoxicity specifically, three near-term opportunities appear particularly tractable. First, classifier training on existing TGx-DDI datasets could generate predictive models capable of ranking novel antibiotic candidates by genotoxic liability during preclinical development. The recent integration of in silico toxicity prediction platforms (Vega Hub and Toxtree) with conventional cytogenetic endpoints for glycopeptide antibiotics demonstrates the practical feasibility of such hybrid frameworks for hazard ranking across understudied antibiotic classes, particularly where conventional in vivo testing is limited by ethical or logistical constraints [75]. Second, automated γH2AX foci scoring using convolutional neural networks could standardize this assay across laboratories and accelerate biomonitoring throughput by one to two orders of magnitude. Third, the integration of pharmacoepidemiologic databases with cytogenetic biobank samples through ML-driven analysis could identify rare adverse outcomes that conventional epidemiology cannot detect.

AI predictions are only as reliable as their training data. The current TGx-DDI training set, for instance, is heavily weighted toward established carcinogens and standard reference chemicals; antibiotic-specific training data are relatively limited. Algorithmic hazard predictions should be regarded as hypothesis-generating screens, not as substitutes for empirical validation. Regulatory acceptance of AI-derived hazard assessments is advancing, but each domain of application requires its own validation.

### 10.4. Single-Cell Genotoxicology

Single-cell whole-genome sequencing, single-cell γH2AX imaging, and single-cell ATAC-seq permit resolution of cell-to-cell heterogeneity in damage burden, repair fidelity, and chromatin accessibility that bulk assays necessarily average away [143]. For antibiotic genotoxicity, the most consequential application is identification of high-risk cell subpopulations within heterogeneous tissues, such as renal proximal tubule progenitor subsets with elevated aminoglycoside susceptibility or cochlear hair cell developmental stages with differential ferroptotic sensitivity. Single-cell mutational signatures (analogous to those developed for environmental and chemotherapeutic exposures by the COSMIC and Mutographs consortia) could in principle identify class-specific antibiotic mutational fingerprints in patient-derived samples, providing direct mechanistic evidence of in vivo genotoxic action [144]. This application is technically feasible today, though analytical pipelines for distinguishing antibiotic-specific signatures from background mutational processes remain to be developed. Cost remains substantial and is a practical limitation for cohort-scale application.

### 10.5. Toward Genotoxically Informed Prescribing

The convergence of the technologies surveyed above might support, within a five-to-ten-year horizon, a precision-medicine framework for genotoxically informed antibiotic selection in high-risk populations. Several components are already mature: pharmacogenomic genotyping is clinically deployable through commercial laboratories and integrated electronic health records; baseline biomarker assessment is feasible from sub-milliliter blood samples; therapeutic drug monitoring is routine for aminoglycosides and increasingly available for other classes; and clinical decision-support algorithms can integrate these data streams at the point of care.

The integrative step that remains is the development of evidence-based prescribing algorithms that incorporate antibiotic class-specific genotoxic burden alongside efficacy, pharmacokinetics, and resistance considerations. In pediatric oncology, neonatal intensive care, pregnancy, and chronic prophylactic contexts, such algorithms could plausibly support genotoxically informed antibiotic selection within the next decade. Whether they will requires sustained investment in cohort generation, biomarker validation, and algorithm development. The historical record of precision-medicine translation in oncology suggests that timelines for clinical implementation are typically longer than initial estimates.

### 10.6. Unresolved Controversies

Several controversies remain open. The magnitude of mammalian ROS contribution to bactericidal antibiotic toxicity, originally proposed by Kohanski and Kalghatgi, has been both supported and challenged by subsequent work and is unlikely to be fully resolved without more sensitive in vivo ROS detection methods. The clinical relevance of in vitro genotoxic signals at supratherapeutic concentrations is debated; tissue accumulation closes some but not all of the in vitro/plasma gap. The carcinogenic risk attributable to chronic or repeated fluoroquinolone exposure is mechanistically nontrivial but epidemiologically unclear, and the available cohort and case-control studies have not provided consistent answers. The translatability of perinatal antibiotic–methylation associations to clinical outcomes is uncertain. Recognition of these unresolved controversies should not paralyze research investment; on the contrary, it argues for prioritizing the studies that could resolve them, particularly large infection-controlled biomonitoring cohorts.

### 10.7. Synthesis: From Discovery to Implementation

The trajectory of antibiotic genotoxicology over the coming decade will be shaped by the simultaneous maturation of four converging trends. Methodological convergence brings together multi-omics, organoid, single-cell, and AI-based approaches. Institutional convergence aligns toxicology, clinical pharmacology, pharmacogenomics, and antimicrobial stewardship around shared problems. Regulatory convergence around NAMs has progressively accepted non-animal hazard data. Conceptual convergence is recognizing host genome integrity as a dimension of drug safety. Each of these trends is already underway. The remaining challenge is integration, that is, the construction of an analytical, regulatory, and clinical infrastructure capable of translating mechanistic insight into safer prescribing for the populations whose health is most at stake.

The argument advanced throughout this review is that this integration is feasible, important, and incomplete. Antibiotic-induced genotoxicity is no longer a sporadic observational signal but a mechanistically tractable, quantitatively measurable, and clinically relevant dimension of drug safety. The cytogenetic toolkit is mature. The molecular mechanisms are increasingly resolved. The vulnerable populations are demographically identifiable. The emerging technologies provide a path toward closing the existing methodological gaps. What is required is institutional commitment to apply these tools systematically, while maintaining appropriate scientific humility about the strength of currently available evidence and the time scale on which translational implementation can realistically be achieved.

## 11. Conclusions

Antibiotic-induced genotoxicity is mechanistically real, biologically heterogeneous, and incompletely characterized at the clinical level. The evidence synthesized in this review converges on a coherent mechanistic architecture organized around three intersecting off-target axes: eukaryotic topoisomerase II interference, mitochondrial dysfunction, and inflammation-coupled redox stress. These axes are not independent. Mitochondrial damage amplifies reactive oxygen species that exacerbate topoisomerase-mediated lesions. Inflammatory cytokines released by damaged cells further escalate oxidative burden. Oxidatively modified repair proteins close a feed-forward loop in which initial injury becomes progressively harder to resolve. The proximal lesion spectrum is correspondingly compact: double-strand breaks, oxidatively modified bases, replication-fork collapse, and chromosomal mis-segregation, each accessible through complementary cytogenetic and molecular assays whose convergent application provides the most defensible evidentiary standard.

The translational dimension of this synthesis warrants careful calibration. Pediatric, geriatric, pregnant, and oncology populations represent biologically distinct susceptibility strata in which developmental pharmacokinetics, immature or declining DNA repair, polypharmacy, antioxidant insufficiency, and pharmacogenomic variation may collectively narrow the genotoxic margin. The pediatric biomonitoring deficit is, by quantitative measure, the largest single evidence gap in the field, and it is technologically tractable: minimal-volume γH2AX methods, opportunistic biobanking, and multi-center pediatric oncology and neonatology networks together delineate a realistic pathway to closure within the next decade. The methodological limitations that currently constrain causal inference (supratherapeutic in vitro dosing, infection as confounder, inter-laboratory assay variability, reliance on immortalized cell lines, and short exposure durations) are similarly addressable through PBPK-integrated study design, paired sampling, primary or iPSC-derived models, and the standardization frameworks now emerging from MIRCA, HUMN, and OECD harmonization.

The emerging technological repertoire of multi-omics integration, microphysiological systems, single-cell genotoxicology, AI-assisted hazard prediction, and pharmacogenomic stratification could in principle support a precision-medicine framework for genotoxically informed antibiotic selection within a realistic time horizon. Several caveats apply. The clinical-endpoint evidence base remains underdeveloped for most antibiotic classes outside metronidazole and fluoroquinolones. Strong claims about carcinogenic risk, transgenerational epigenetic programming, or pediatric-specific susceptibility are mechanistically motivated but not yet empirically established for most exposures. Stewardship recommendations should be informed by, rather than driven exclusively by, mechanistic considerations.

The central argument of this review is, therefore, both a scientific synthesis and a measured policy proposition. Antibiotic genotoxicity is a quantifiable and mechanistically tractable dimension of drug safety. Antimicrobial stewardship, historically framed as the protection of antibiotic effectiveness against resistance, would benefit from incorporating the protection of host genome integrity into its conceptual scope, particularly in vulnerable populations. The mechanistic case for this expansion is well-supported. The translational tools are increasingly available. The populations are demographically identifiable. The remaining task is the construction of an evidence base sufficient to support the policy reframing that the mechanistic literature already invites.

## Figures and Tables

**Table 1:** Principal antibiotic classes considered in this review, with bacterial targets, established eukaryotic off-target liabilities, and dominant proximal genotoxic mechanism.

Class	Representative Agents	Bacterial Target	Eukaryotic Off-Target	Dominant Proximal Mechanism	Section
Fluoroquinolones	Ciprofloxacin, levofloxacin, moxifloxacin	DNA gyrase, Topo IV	Topo IIα/β; mitochondrial Topo II	Trapped cleavage complexes → DSB; mtDNA damage	Section 2.1
Nitroimidazoles	Metronidazole, tinidazole, ornidazole	Bioreductive activation in anaerobes	Hypoxic mammalian niches	Nitro-radical anion → DNA adducts, SSB, AP sites	Section 2.2
Aminoglycosides	Gentamicin, amikacin, tobramycin, streptomycin	30S ribosome (A-site)	Mitoribosome (12S rRNA); iron complexes	Mitoribosomal inhibition; Fenton-type •OH	Section 2.3
Macrolides	Azithromycin, clarithromycin, erythromycin	50S ribosome (peptide exit tunnel)	39S mitoribosome	ETC subunit depletion; ΔΨm collapse; ROS	Section 2.4
β-Lactams	Amoxicillin, ceftriaxone, meropenem	Penicillin-binding proteins	No direct eukaryotic target	Microbiome dysbiosis → bile-acid genotoxicity	Section 2.5
Tetracyclines	Doxycycline, minocycline, tetracycline	30S ribosome (aminoacyl-tRNA site)	Mitochondrial translation; MMPs	Chronic mtDNA depletion; secondary ROS	Section 2.6
Glycopeptides	Vancomycin, teicoplanin	D-Ala-D-Ala of peptidoglycan	No obvious eukaryotic counterpart	Measurable cytotoxic/genotoxic signals reported	Section 2.7
Glycylcyclines	Tigecycline	Modified 30S ribosomal binding	Shared with tetracyclines	Mitochondrial translation inhibition	Section 2.7

Topo, topoisomerase; DSB, double-strand break; SSB, single-strand break; AP, apurinic/apyrimidinic; ETC, electron transport chain; ΔΨm, mitochondrial membrane potential; ROS, reactive oxygen species; mtDNA, mitochondrial DNA; MMP, matrix metalloproteinase.

**Table 2:** Central evidence summary: key studies on antibiotic-induced genotoxicity discussed in this review.

Antibiotic	Class	Experimental Model	Dose/Concentration	Duration	Genotoxic Endpoint	Main Result	Clinical Relevance	Evidence Type	Principal Limitation
Ciprofloxacin	Fluoroquinolone	HepG2 cells	50–200 μM	NR	mtDNA cleavage	Concentration-dependent mtDNA cleavage [23]	200 mg oral Cmax ≈3.6 μM; 500 mg ≈6–12 μM; supratherapeutic	In vitro	Immortalized cell line; supratherapeutic concentrations
Ciprofloxacin	Fluoroquinolone	HeLa cells	Similar range (50–200 μM)	NR	mtDNA replication impairment	mtDNA replication inhibition [12]	Supratherapeutic; tissue accumulation 3–5× narrows gap	In vitro	Single cell line; no DNA strand break measurement
Ciprofloxacin	Fluoroquinolone	TK6 cells	≥25 μM	NR	γH2AX foci	DSB induction from 25 μM [38]	Approaching tissue concentrations with repeated dosing	In vitro	Single cell line
Ciprofloxacin	Fluoroquinolone	Human lymphocytes	NR	NR	MN, comet, SCE	Positive CBMN, comet, and SCE signals [39]	NR	In vitro (human cells)	Concentration and duration details require verification
Metronidazole	Nitroimidazole	Human lymphocytes	Therapeutic course	7–10 days	MN, comet, SCE	Reproducible CBMN, comet, SCE signals [9,39,40]	Standard therapeutic exposure	Human biomonitoring	Infection as confounder; no infection-only controls
Metronidazole	Nitroimidazole	In vitro/in vivo	NR	NR	DNA adducts, SSB	Covalent binding to guanine (N-2, C-8) and adenine	Bioactivation favored under hypoxia; limited in normoxic tissue	In vitro + animal	Oxygen futile cycling limits activation in aerobic host cells
Gentamicin	Aminoglycoside	Renal proximal tubule cells	≥25 μM	NR	8-OHdG, mitochondrial dysfunction	ROS-mediated oxidative DNA damage	Renal accumulation 30–50× plasma; estimated >250 μM in cortex	In vitro + animal	Tissue concentrations estimated from ratios, not directly measured
Gentamicin	Aminoglycoside	Cochlear hair cells	NR	NR	mtDNA damage, 8-OHdG	Mitochondrial and oxidative DNA damage	Cochlear accumulation via megalin/cubilin	In vitro + animal	Endpoint is organ toxicity; DNA damage measured as secondary outcome
Azithromycin	Macrolide	Cardiomyocytes	NR	Hours	Mitochondrial dysfunction, ROS, 8-OHdG	Mitochondrial superoxide bursts; elevated 8-OHdG	Tissue:plasma ratio 10–100×; intracellular accumulation	In vitro	Cell types selected for high mitochondrial content; unclear if effect occurs at clinical concentrations
Amoxicillin	β-Lactam	Intestinal model/in vivo	NR	NR	Indirect—microbiome-bile-acid axis	Dysbiosis-mediated indirect pathway proposed	Widely prescribed at standard doses	In vitro + animal	Indirect mechanism; no direct DNA damage measurement
Doxycycline	Tetracycline	Multiple cell types	NR	Chronic	mtDNA copy number, ETC function	Chronic mtDNA depletion and late ROS	Phototoxicity-mediated ROS component	In vitro + animal	Chronic exposure models; limited genotoxic endpoint data

NR = not reported in the cited source or not available from the manuscript text. MN = micronuclei; SCE = sister chromatid exchange; DSB = double-strand break; SSB = single-strand break; ETC = electron transport chain. Entries are based on studies discussed in this review; the table is not exhaustive. Dose/concentration and duration values marked NR should be verified by the authors against original source publications. Clinical relevance assessments refer to the relationship between in vitro test concentrations and clinically achievable tissue exposure and do not imply established clinical risk. [AUTHOR INPUT REQUIRED: Authors should verify all entries against original sources and add additional key studies as appropriate.].

**Table 3:** Adverse outcome pathway (AOP) framework for antibiotic-induced genotoxicity, organized around three convergent off-target axes.

AOP Component	Topo II Axis	Mitochondrial Axis	Inflammation–Redox Axis
Molecular initiating event (MIE)	FQ binding to Topo IIα/β	Mitoribosome inhibition; mtTopo II inhibition	Microbiome dysbiosis; direct ROS
Key event 1	Cleavage complex stabilization	ETC subunit depletion; complex I/IV destabilization	Bile-acid metabolism shift; NF-κB activation
Key event 2	Replication/transcription collision → DSB	ΔΨm collapse; mitochondrial ROS burst	NLRP3 inflammasome; IL-1β/IL-6/TNF-α
Key event 3	ATM activation; γH2AX foci formation	mtDNA damage; cytosolic mtDNA release	Innate immune recruitment; NOX2/iNOS burst
Key event 4	Repair pathway choice (HR/NHEJ/MMEJ)	cGAS-STING activation; type I IFN	Paracrine genotoxic stress on neighbors
Adverse outcome	Chromosomal translocation; MN; carcinogenic risk	Apoptosis; ferroptosis; tissue degeneration	Chronic genotoxic microenvironment
Principal class	Fluoroquinolones	Aminoglycosides; macrolides; tetracyclines	β-Lactams (indirect); bactericidal agents
Detection assay	γH2AX, comet (neutral), CBMN-Cyt	MitoSOX, ΔΨm, 8-OHdG, mtDNA copy number	Cytokine panel; 8-OHdG; lipid peroxidation

FQ, fluoroquinolone; HR, homologous recombination; NHEJ, non-homologous end joining; MMEJ, microhomology-mediated end joining; MN, micronucleus; NOX2, NADPH oxidase 2; iNOS, inducible nitric oxide synthase; cGAS, cyclic GMP-AMP synthase; STING, stimulator of interferon genes; IFN, interferon.

**Table 4:** The evidence-strength matrix for the prioritized antibiotic classes across principal cytogenetic and molecular endpoints. Strength designations reflect both the magnitude and reproducibility of the available data.

Class	γH2AX/DSB	Comet/SSB	MN/CBMN-Cyt	8-OHdG/Oxidative	mtDNA Damage	Human Biomonitoring	Overall Strength
Fluoroquinolones	Strong	Strong	Moderate	Moderate	Strong	Moderate	Strong
Nitroimidazoles	Moderate	Strong	Strong	Moderate	Weak	Moderate	Strong
Aminoglycosides	Moderate	Moderate	Weak	Strong	Strong	Weak	Moderate
Macrolides	Moderate	Weak	Weak	Moderate	Moderate	Weak	Moderate
β-Lactams	Weak	Weak	Weak	Moderate (indirect)	Weak	Weak	Weak
Tetracyclines	Weak	Moderate	Weak	Moderate	Strong (chronic)	Weak	Moderate (chronic)
Glycopeptides (teicoplanin)	Moderate (recent)	Moderate (recent)	Moderate (recent)	Not characterized	Not characterized	Absent	Emerging
Glycylcyclines (tigecycline)	Not characterized	Strong (in vivo bladder)	Not characterized	Not characterized	Plausible (parent class)	Absent	Emerging

Strength categories were assigned using the following qualitative framework. Strong: three or more independent positive studies with consistent findings across at least two experimental models or assay types, with at least one study at clinically achievable concentrations and no predominant negative findings. Moderate: reproducible positive signals from at least two independent studies, but limited to a single experimental system, or inconsistency between in vitro and in vivo results, or positive results only at supratherapeutic concentrations. Weak: isolated positive findings, predominant negative results, or positive results only at concentrations far exceeding therapeutic exposure. Emerging: recent positive findings, typically from single studies published within the past 3–5 years, that have not yet been replicated. These criteria were developed by the authors as a qualitative interpretive framework and do not constitute a validated scoring system. Designations reflect strength of available evidence rather than the magnitude of clinical risk.

**Table 5:** Principal cytogenetic, oxidative, and mitochondrial biomarkers used in antibiotic–genotoxicity studies, with OECD status, biological dimension, strengths, and limitations.

Assay/Biomarker	OECD TG	Endpoint Detected	Strengths	Limitations
CBMN-Cyt	487 (in vitro)	Micronuclei, NPBs, NBUDs (clastogenic + aneugenic)	Captures multiple endpoints; HUMN database; scalable	Insensitive to non-mitotic lesions; cytotoxicity confounding
Alkaline comet	489 (in vivo)	SSB + DSB; oxidized bases (with Fpg)	High sensitivity; single-cell resolution; low input	Inter-laboratory variability; multiple readouts
Neutral comet	Not formal	DSB selective	DSB specificity; quantitative	Less standardized than alkaline
Chromosomal aberration	473 (in vitro); 475 (in vivo)	Breaks, dicentrics, rings, exchanges	Regulatory benchmark; predicts cancer risk	Labor-intensive; expert scoring; lower sensitivity
Sister chromatid exchange	—	Recombinational activity	Historical comparability with 1970s–1980s surveys	Ambiguous mechanistic interpretation
γH2AX foci	Not formal	DSB initiation; single-DSB sensitivity	Gold-standard DSB marker; blood-drop method available	Apoptotic confounding; threshold variability
TUNEL	—	Apoptotic DNA fragmentation	Distinguishes terminal apoptosis	Downstream marker, not primary genotoxicity
8-OHdG (UHPLC-MS/MS)	—	Oxidative DNA damage	Quantitative; urinary; gold standard	ELISA cross-reactivity; preanalytical confounders
MitoSOX (O_2_•^−^)	—	Mitochondrial superoxide	Real-time imaging in live cells	Probe oxidation kinetics; ΔΨm-dependent loading
MDA/TBARS	—	Lipid peroxidation	Established methodology; broad applicability	TBARS non-specific; HPLC preferred
GSH/GSSG ratio	—	Cellular thiol redox state	Direct mechanistic interpretation	Sample handling critical (rapid GSH oxidation)
ΔΨm (JC-1, TMRM)	—	Mitochondrial membrane potential	Functional readout; flow-compatible	Probe distribution depends on loading conditions
mtDNA copy number	—	Mitochondrial damage/depletion	Sensitive to chronic exposure	Tissue heterogeneity; baseline variability
mtDNA 4977-bp deletion	—	Chronic mitochondrial damage	Long-term cumulative exposure marker	Age-dependent baseline; correction needed
TGx-DDI biomarker	Regulatory consideration	DDR transcriptional signature (64 genes)	Mechanistic granularity; high accuracy	Antibiotic-specific validation pending

OECD TG, OECD Test Guideline; SSB, single-strand break; DSB, double-strand break; NPB, nucleoplasmic bridge; NBUD, nuclear bud; CBMN-Cyt, cytokinesis-block micronucleus cytome; UHPLC-MS/MS, ultra-high-performance liquid chromatography–tandem mass spectrometry; HUMN, HUman MicroNucleus project; DDR, DNA damage response; TGx-DDI, transcriptomic biomarker for DNA damage-inducing chemicals; MDA, malondialdehyde; TBARS, thiobarbituric acid reactive substances; GSH/GSSG, reduced/oxidized glutathione ratio; JC-1, JC-1 mitochondrial probe; TMRM, tetramethylrhodamine methyl ester; ΔΨm, mitochondrial membrane potential.

**Table 6:** Human biomonitoring studies and gaps for antibiotic-induced genotoxicity across vulnerable populations.

Population	Available Evidence	Principal Assays Used	Critical Gap
Adult patients on metronidazole	Moderate (small cohorts)	CBMN-Cyt; comet; SCE	Lack of infection-controlled designs
Adult patients on fluoroquinolones	Limited and inconsistent	CBMN-Cyt; γH2AX; comet	No large multi-center cohorts
Adult patients on β-lactams	Minimal (typically negative)	CBMN-Cyt; comet	Microbiome-mediated effects rarely assessed
Pediatric (general)	<5% of the biomonitoring literature	CBMN-Cyt (limited)	Minimal-volume γH2AX biobanking needed
Neonates/NICU	Essentially absent	—	Critical gap; cord-blood biobanking feasible
Pregnant women	Essentially absent	—	Ethical/regulatory barriers; opportunistic sampling possible
Pediatric oncology	Confounded by chemotherapy	γH2AX (chemo-confounded)	Factorial designs needed
Cystic fibrosis (chronic AG)	Limited	Audiometric; renal biomarkers	Cytogenetic biomonitoring absent
Elderly polypharmacy	Minimal	—	Combinatorial exposure framework needed
Pharmaceutical industry workers	Available for several classes	CBMN-Cyt; SCE	Translational extrapolation to therapeutic exposure unclear

NICU, neonatal intensive care unit; AG, aminoglycoside; SCE, sister chromatid exchange. The pediatric biomonitoring deficit represents the largest single evidence gap in the field; technologically tractable solutions are available (the γH2AX blood-drop method [113]; opportunistic biobanking) but require coordinated institutional commitment.

**Table 7:** Principal methodological gaps in antibiotic–genotoxicity research and tractable approaches to closure.

Gap	Current State	Tractable Approach	Realistic Time Horizon
In vitro vs. clinical exposure mismatch	Supratherapeutic in vitro doses common	PBPK-integrated study design; tissue-accumulation modeling	2–5 years
Reliance on immortalized cell lines	HeLa, V79, CHO, TK6, HepG2 dominate	Primary cells; iPSC-derived models; organoids	5–10 years
Acute vs. chronic exposure paradigms	Short (3–24 h) exposures dominate	Chronic-exposure microphysiological systems	5–10 years
Infection-as-confounder	Rare paired-sampling designs	Pre-/on-/post-treatment with infection-only controls	2–5 years
Inter-laboratory assay variability	Substantial heterogeneity persists	Ring-trial validation; MIRCA/HUMN expansion	2–5 years
Pediatric biomonitoring deficit	<5% of the cytogenetic literature	Minimal-volume γH2AX; cord-blood biobanking	5–10 years
Pregnancy biomonitoring	Essentially absent	Opportunistic prenatal sampling; ethics-cleared cohorts	5–10 years
Mixed-drug interactions	Almost uninvestigated	Factorial in vitro designs; pharmacoepi databases	2–5 years
Microbiome-mediated genotoxicity	Mechanistic but not quantified	Gnotobiotic models; gut-on-chip platforms	5–10 years
Pharmacogenomic stratification	Small, underpowered studies	Multi-center genotype-stratified cohorts	5–10 years

PBPK, physiologically-based pharmacokinetic modeling; iPSC, induced pluripotent stem cell; MIRCA, Minimum Information for Reporting on the Comet Assay; HUMN, HUman MicroNucleus project.

**Table 8:** Emerging methodological technologies for antibiotic–genotoxicity research, with current readiness and principal limitations.

Technology	Current Readiness	Antibiotic-Relevant Application	Principal Limitations
TGx-DDI transcriptomic biomarker	Regulatory consideration	Class-specific signature discovery	Antibiotic-specific validation pending
Multi-omics integration	Mechanistic discovery phase	Chronic-exposure phenotyping	Cost; analytical complexity; harmonization
Microphysiological systems (organ-on-chip)	Mechanistic discovery; some regulatory acceptance	Tissue-specific PK; pediatric/pregnancy proxies	Standardization; throughput; cost
iPSC-derived organoids	Discovery to early translation	Patient-specific susceptibility modeling	Reproducibility; cost; cell-source variability
High-content γH2AX imaging	Mature for screening	Automated biomonitoring throughput	Threshold standardization across platforms
Single-cell genotoxicology	Mature for discovery	Subpopulation risk identification	Cost; cohort-scale unfeasibility currently
Single-cell mutational signatures	Methodologically feasible	In vivo antibiotic mutational fingerprints	Background-signature separation; bioinformatics pipelines
AI/ML hazard prediction (QSAR, deep learning)	Discovery to regulatory consideration	Pre-clinical candidate ranking	Training-data limitations; antibiotic-specific data sparse
Automated foci/comet scoring (CNN-based)	Mature for some platforms	Biomonitoring throughput	Cross-platform comparability
Pharmacogenomic stratification	Clinically deployable	Risk-stratified prescribing	Antibiotic–genotoxicity evidence base limited

PK, pharmacokinetics; iPSC, induced pluripotent stem cell; QSAR, quantitative structure-activity relationship; CNN, convolutional neural network; ML, machine learning. Time horizons for clinical implementation typically exceed initial estimates.

## Data Availability

No new data were created or analyzed in this study. The literature search strategy supporting the conclusions is described in Section 1.6. Data sharing is not applicable to this article.
